# Correction: Molecular response to the non-lytic peptide bac7 (1–35) triggers disruption of *Klebsiella pneumoniae* biofilm

**DOI:** 10.1371/journal.ppat.1014298

**Published:** 2026-06-09

**Authors:** Robert L. Beckman I. V, Berta Victoria, Flor Z. Santiago, Gabriela N. Echeverria, Bruno V. Pinheiro, Marcelo D.T. Torres, Logan Suits, Shantal Garcia, Paeton L. Wantuch, Cesar de la Fuente-Nunez, Prahathees Eswara, David A. Rosen, Renee M. Fleeman

The second author’s name is spelled incorrectly. The correct name is: Berta Victoria. The correct citation is: Beckman I.V RL, Victoria B, Santiago FZ, Echeverria GN, Pinheiro BV, D.T. Torres M, et al. (2025) Molecular response to the non-lytic peptide bac7 (1–35) triggers disruption of Klebsiella pneumoniae biofilm. PLoS Pathog 21(12): e1013437. https://doi.org/10.1371/journal.ppat.1013437

## References

[1] Beckman I V RL, Martinez B, Santiago FZ, Echeverria GN, Pinheiro BV, D T Torres M, et al. Molecular response to the non-lytic peptide bac7 (1-35) triggers disruption of Klebsiella pneumoniae biofilm. PLoS Pathog. 2025;21(12):e1013437. doi: 10.1371/journal.ppat.1013437 41325418 PMC12677791

