# Supplementary figures and images for: Molecular response to the non-lytic peptide bac7 (1–35) triggers disruption of Klebsiella pneumoniae biofilm
Source: PLoS Pathog. 2025 Dec 1;21(12):e1013437. doi: 10.1371/journal.ppat.1013437 (PMC12677791; doi:10.1371/journal.ppat.1013437)

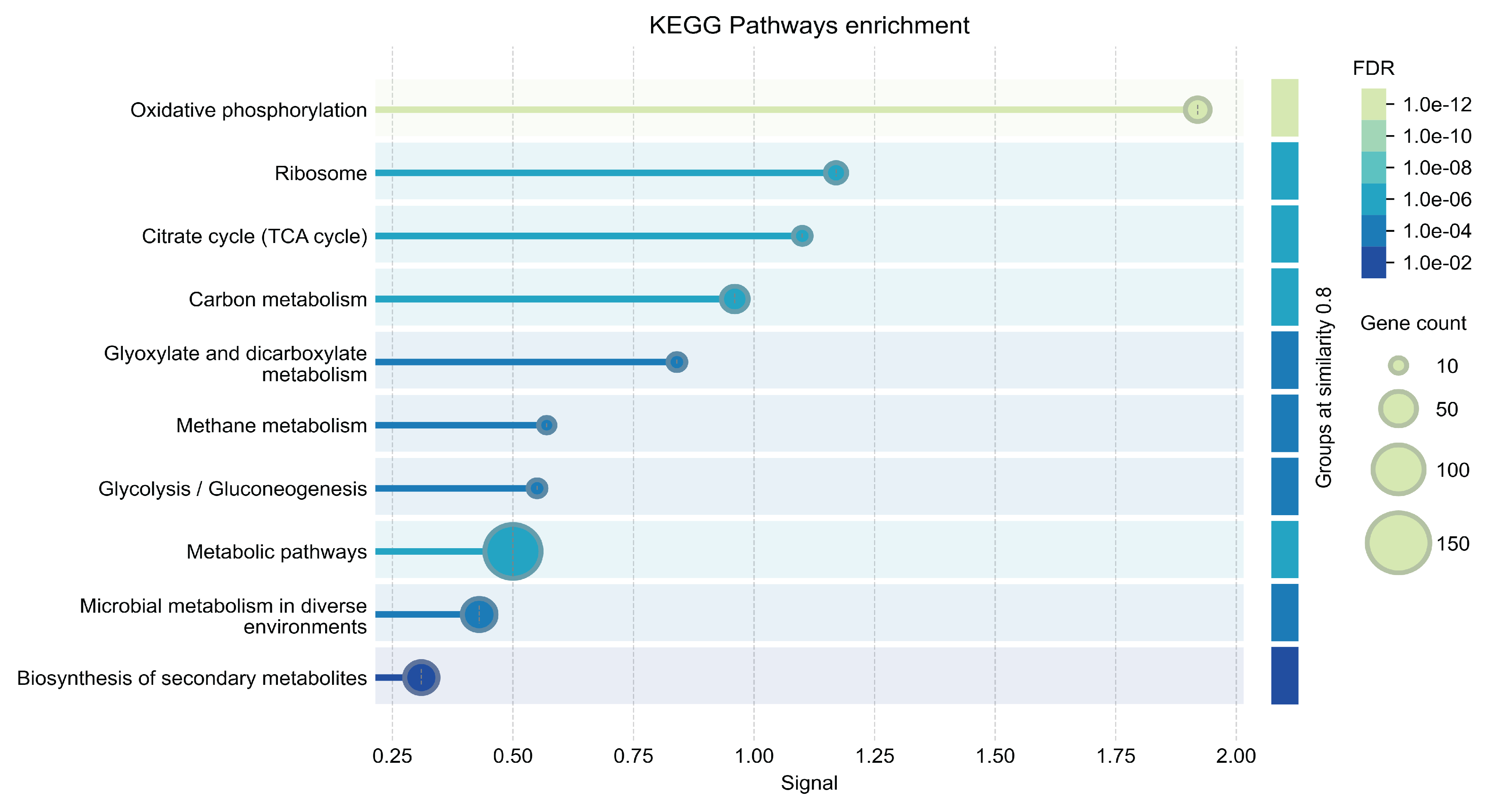

Supplement: S1 Fig — The figure shows the total pathways effected by bac7 (1–35) treatment. Ribosome pathway is upregulated with bac7 treatment compared to the control no treatment sample and all other pathways shown enriched are downregulated with bac7 treatment compared to the control no treatment sample. (TIF) [file ppat.1013437.s001.tif]

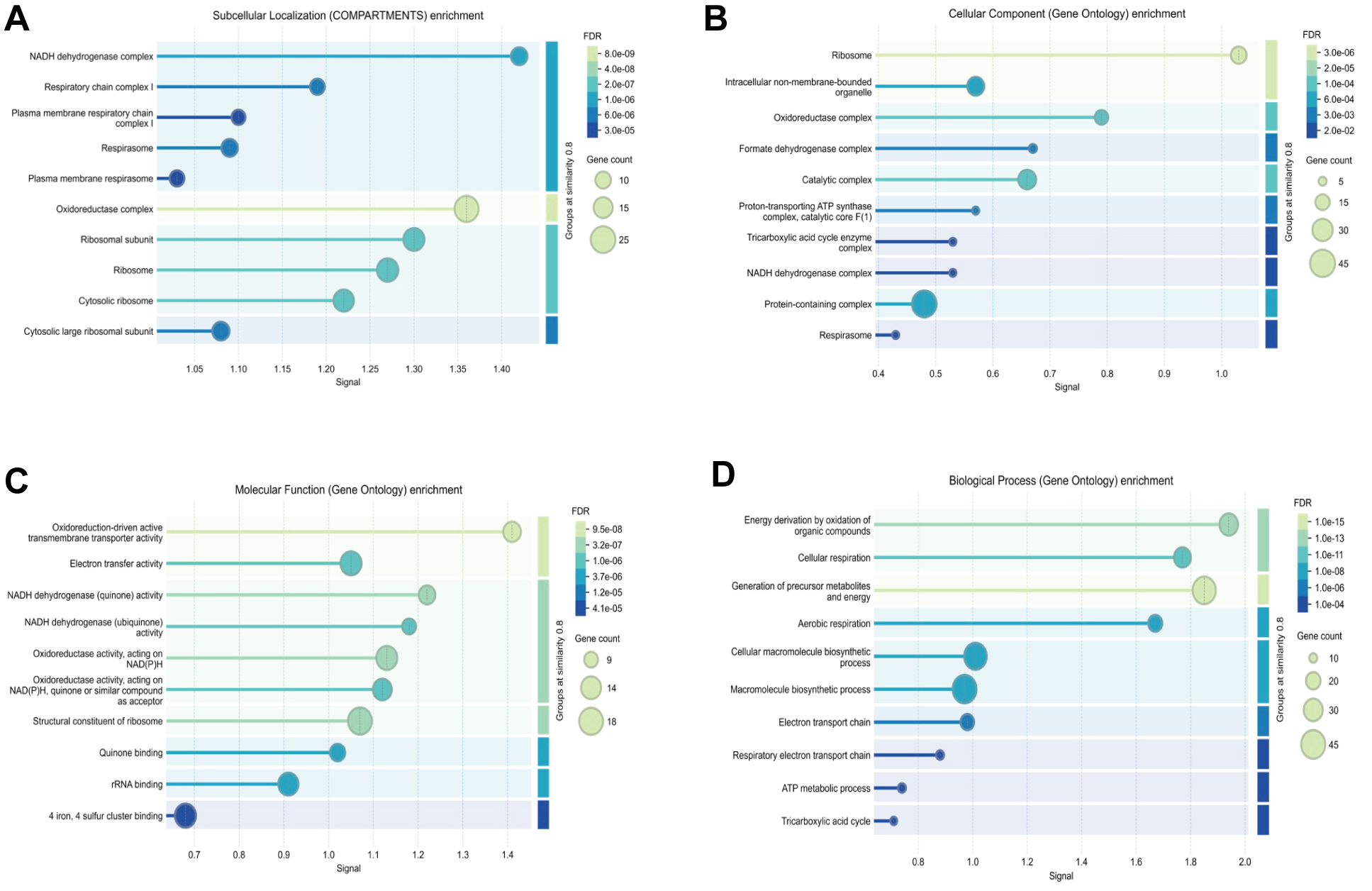

Supplement: S2 Fig — The figures show the gene ontology of the changes associated with bac7 (1–35) treatment when considering subcellular localization (A) cellular component (B), molecular function (C), and biological processes (D). (TIF) [file ppat.1013437.s002.tif]

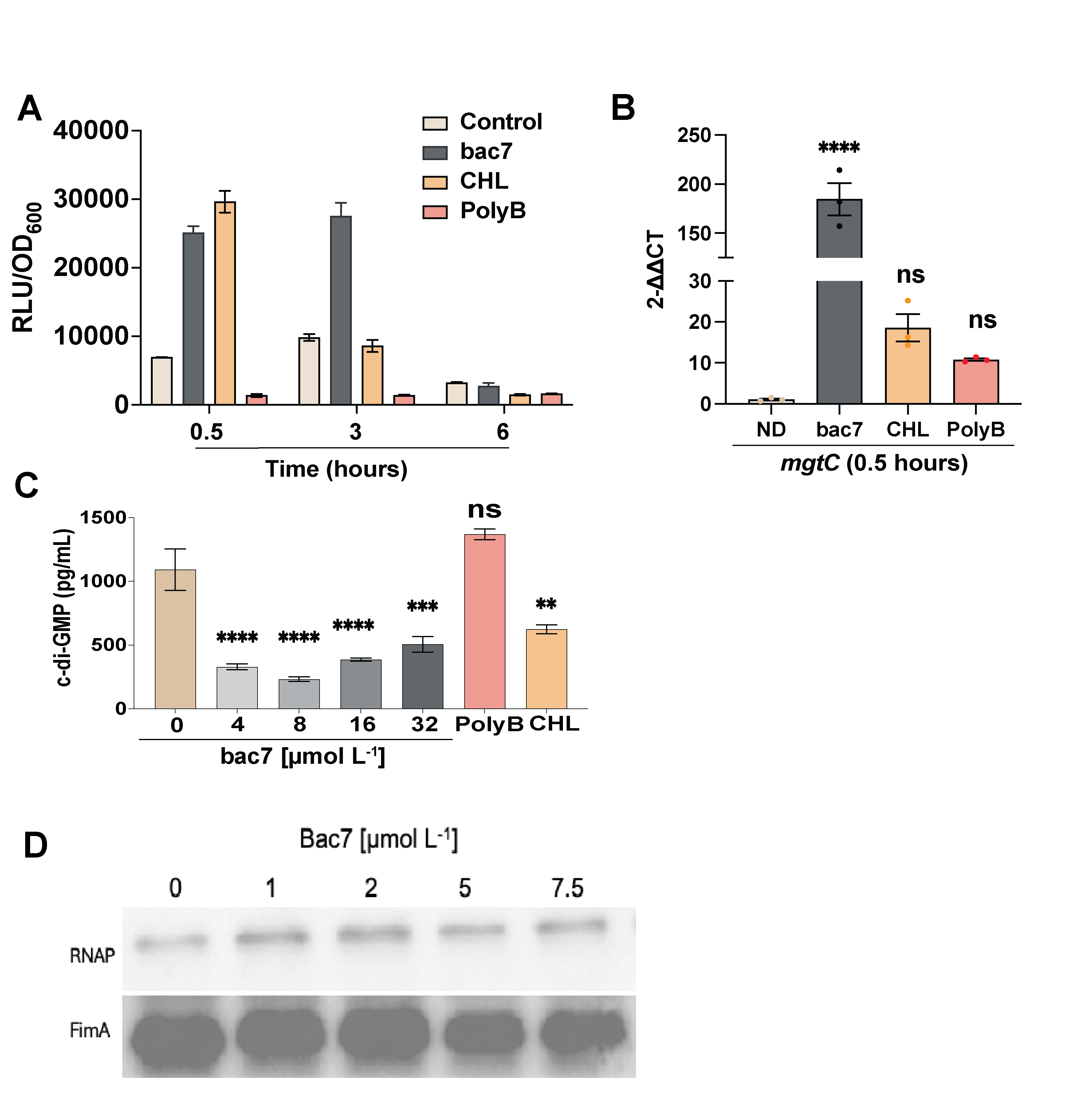

Supplement: S4 Fig — Panel A shows ATP at 30 minutes, 3 hours, and 6 hours with bac7 treatment compared to chloramphenicol and polymyxin B ribosomal binding and lytic peptide controls, respectively. Panel B shows RT-qPCR of mgtC 30 minutes following treatment with bac7 and controls. Panel C shows c-di-GMP quantification following treatment with bac7 for 30 minutes. ATP and c-di-GMP quantification was performed next to the respective ribosomal and lytic controls chloramphenicol (CHL) at 396 µmol L-1, polymyxin B (PolyB) at 25 µmol L-1. One-way ANOVA was used to determine significance for B and C with Dunnett’s correction for multiple comparisons with adjusted p-values shown (asterisks indicate p-values **** < 0.0001, ** < 0.01, and ns > 0.1) and error shown reported as ±SEM. Panel D shows the Immunoblot analysis of K. pneumoniae NTUH K2044 with increasing concentrations of bac7 (1 - 7.5 µmol L-1). (TIF) [file ppat.1013437.s004.tif]

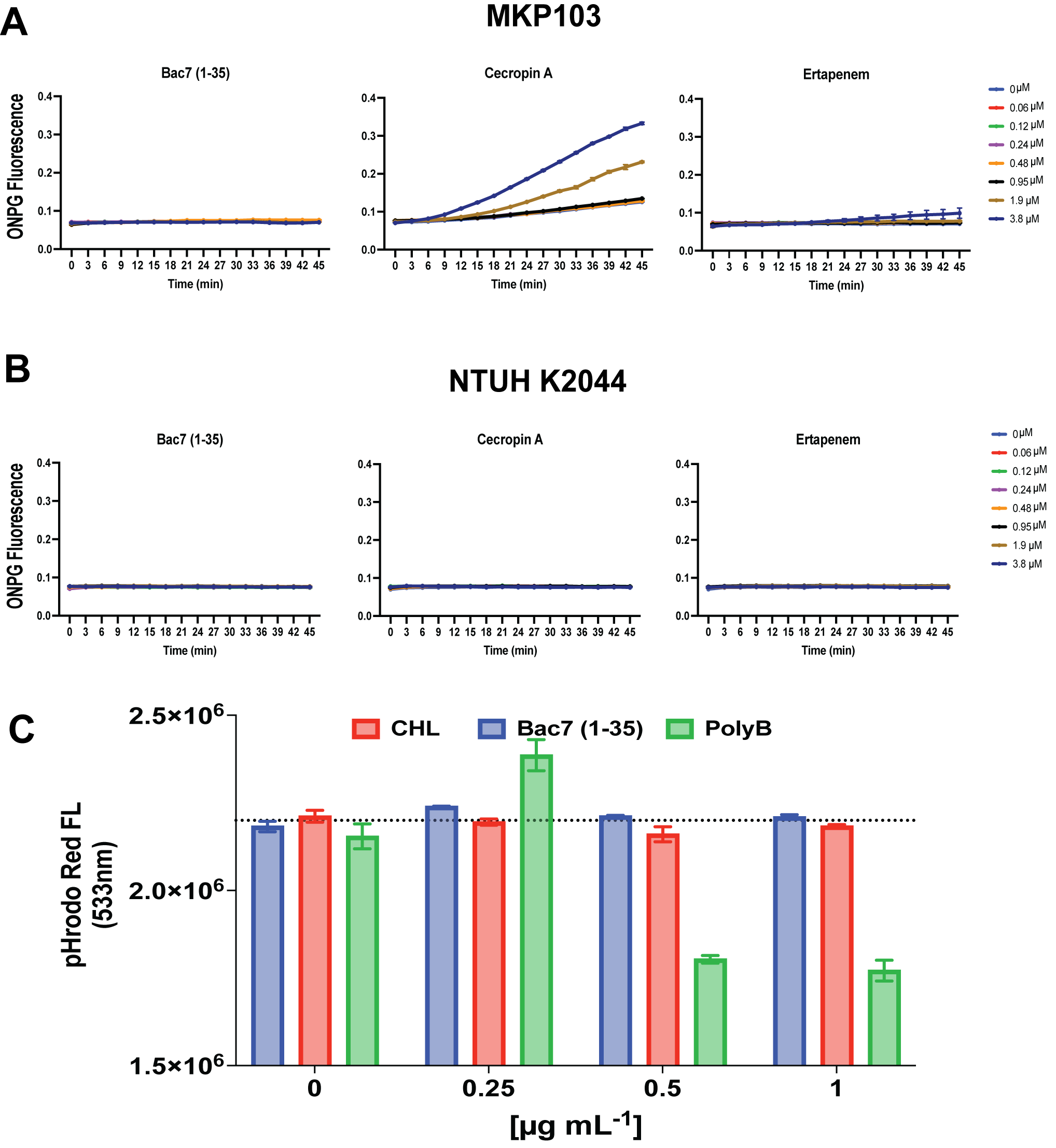

Supplement: S5 Fig — Panel A shows a β-galactosidase leakage assay using o-nitrophenyl-β-d-galactopyranoside (ONPG) in cKp control MKP103 after treatment with bac7 (1–35), positive control cecropin A, and negative control ertapenem. Panel B displays ONPG assay in hvKp NTUH K2044 after treatment with bac7 (1–35), cecropin A, and ertapenem. Normalized fluorescence intensity values were achieved from subtracting blank 96-well plate intensity values by sample values. Panel C shows intracellular pH changes with treatment of chloramphenicol, bac7 (1–35), and polymyxin B. The fluorescence readouts were graphed with error reported as ±SEM. (TIF) [file ppat.1013437.s005.tif]

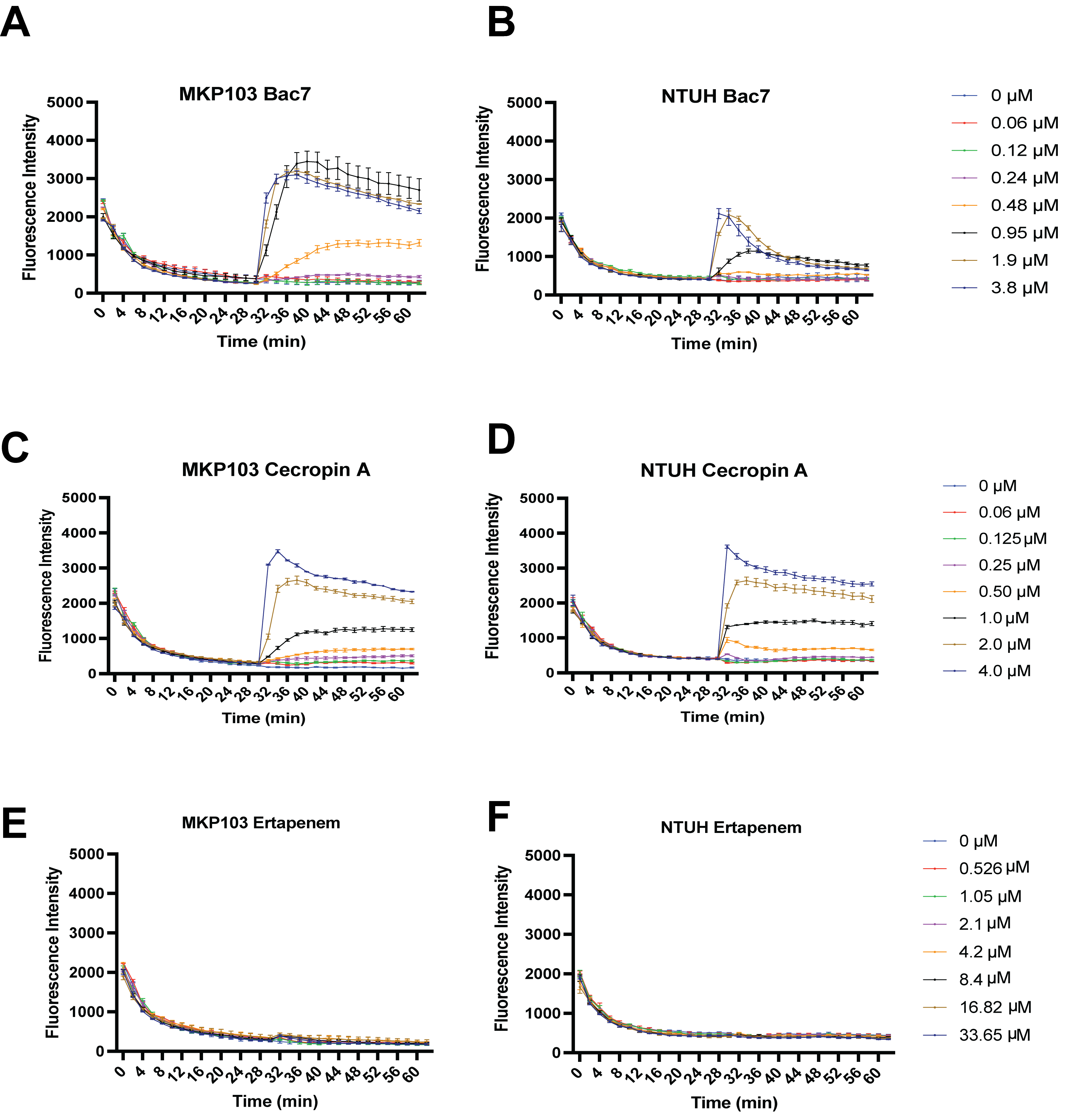

Supplement: S6 Fig — The Figure shows the kinetic depolarization of control strains hvKp NTUH K2044 and cKp MKP103 with bac7 (1–35) (A and B), positive control lytic peptide cecropin A (C and D), and negative control non-lytic ertapenem (E and F). Cationic hydrophobic DiSC3(5) (3,3’-Dipropylthiadicarbocyanine Iodide) dye was used. Readouts were measured in fluorescence intensity and normalized fluorescence intensity values were achieved by subtracting blank wells in 96-well plate from sample wells. Errors were reported as ±SEM. (TIF) [file ppat.1013437.s006.tif]

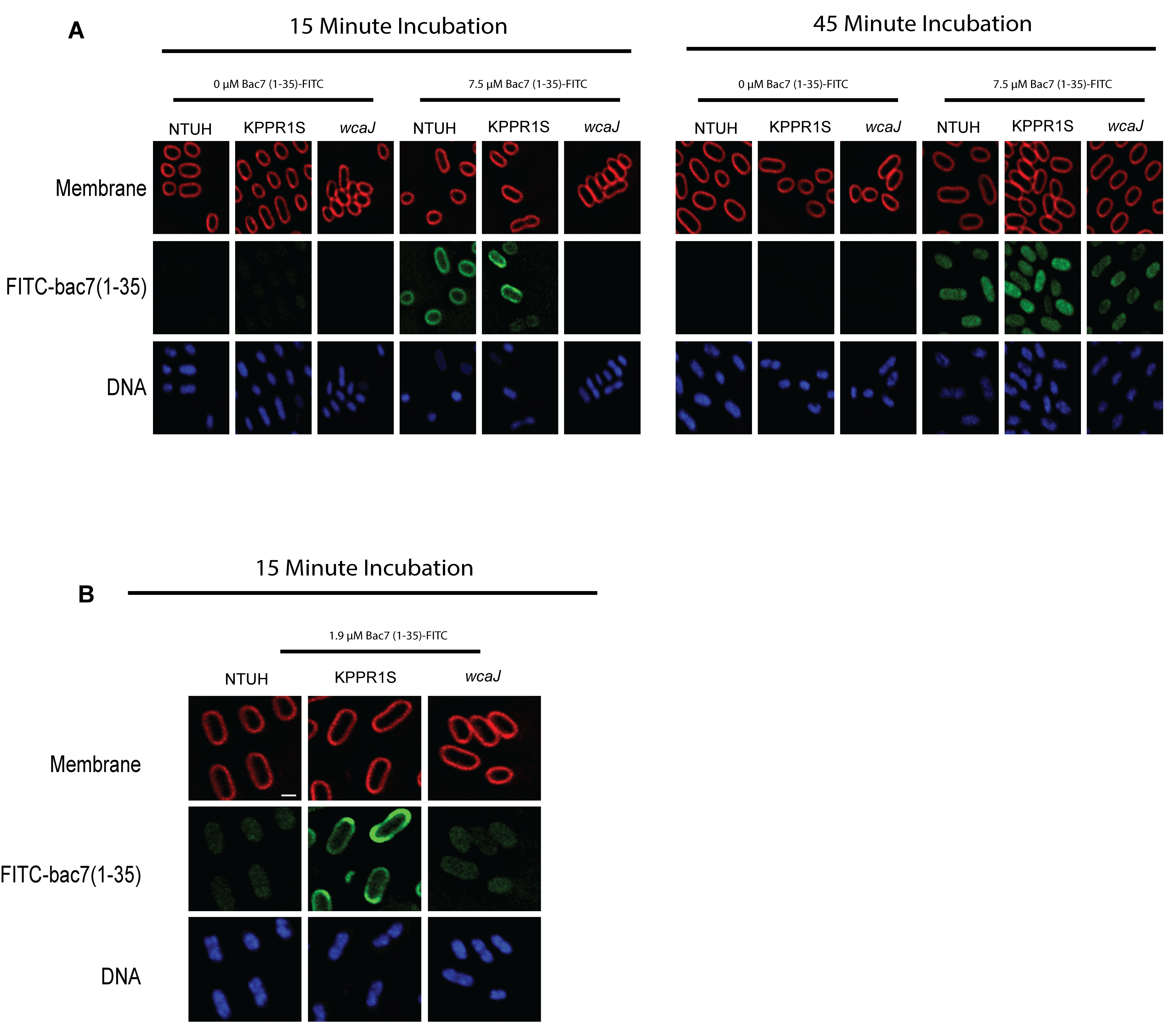

Supplement: S7 Fig — HvKp control strains NTUH K2044 (K1), KPPR1S (K2), and its capsular mutant ΔwcaJ were treated with 0 and 7.5 µmol L-1 and incubated for 15 and 45 minutes (A). Cell membranes were stained with FM4–64 red dye and DAPI blue, fluorescent dye was used to stain the cellular DNA. B shows hvKp control strains NTUH K2044 (K1), KPPR1S (K2), and its capsular mutant ΔwcaJ treated with 1.9 FITC-labeled bac7 (1–35) for 15 and 45 minutes. Membrane and DNA staining described above were utilized. (TIF) [file ppat.1013437.s007.tif]

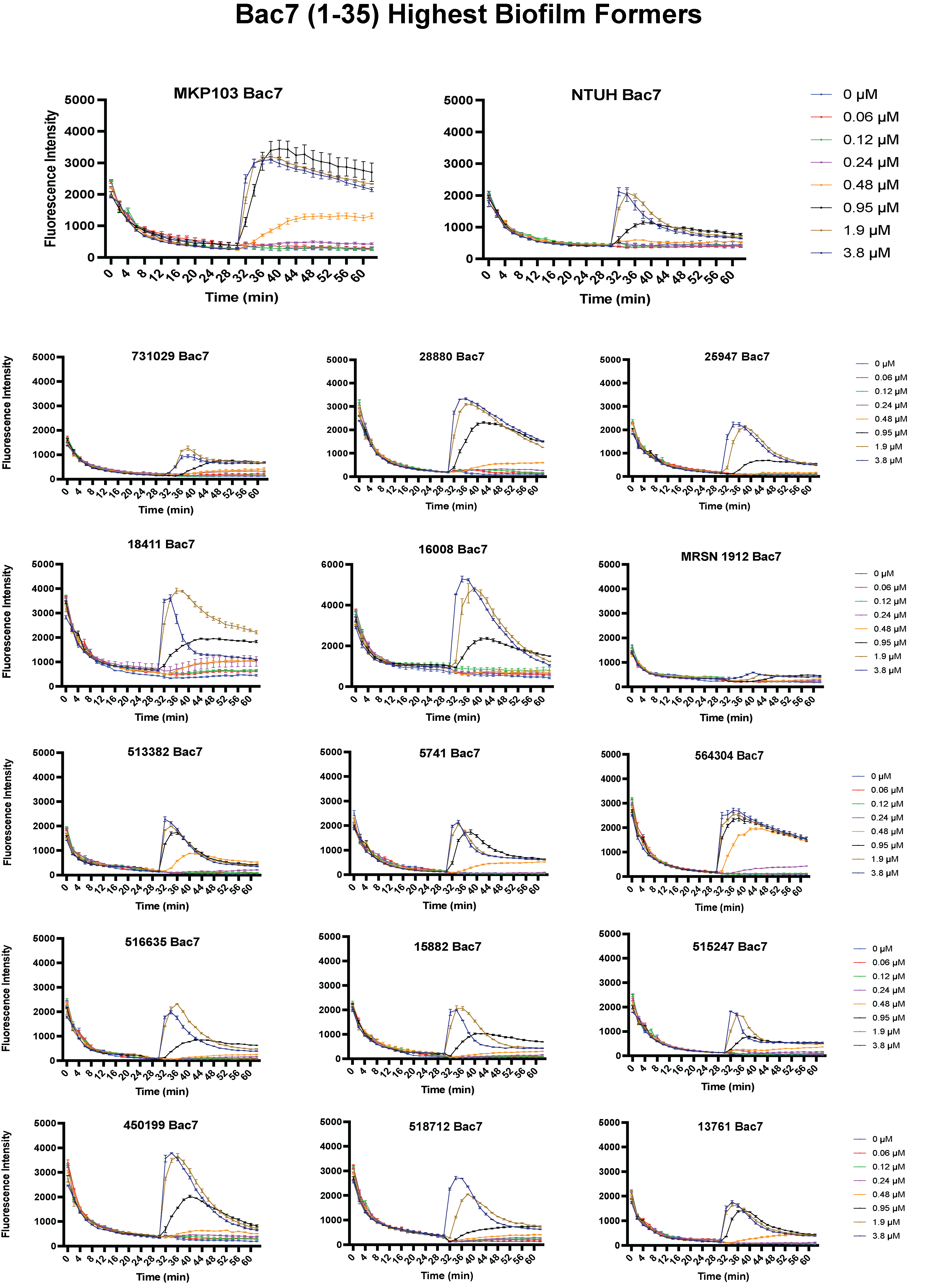

Supplement: S8 Fig — The Figure shows the kinetic depolarization of control strains hvKp NTUH K2044 and cKp MKP103 as well as the highest biofilm-forming isolates in the MRSN diversity panel after treatment with bac7 (1–35) for 30 minutes. Cationic hydrophobic DiSC3(5) (3,3’-Dipropylthiadicarbocyanine Iodide) dye was used. Readouts were measured in fluorescence intensity and normalized fluorescence intensity values were achieved by subtracting blank wells in 96-well plate from sample wells. Errors were reported as ±SEM. (TIF) [file ppat.1013437.s008.tif]

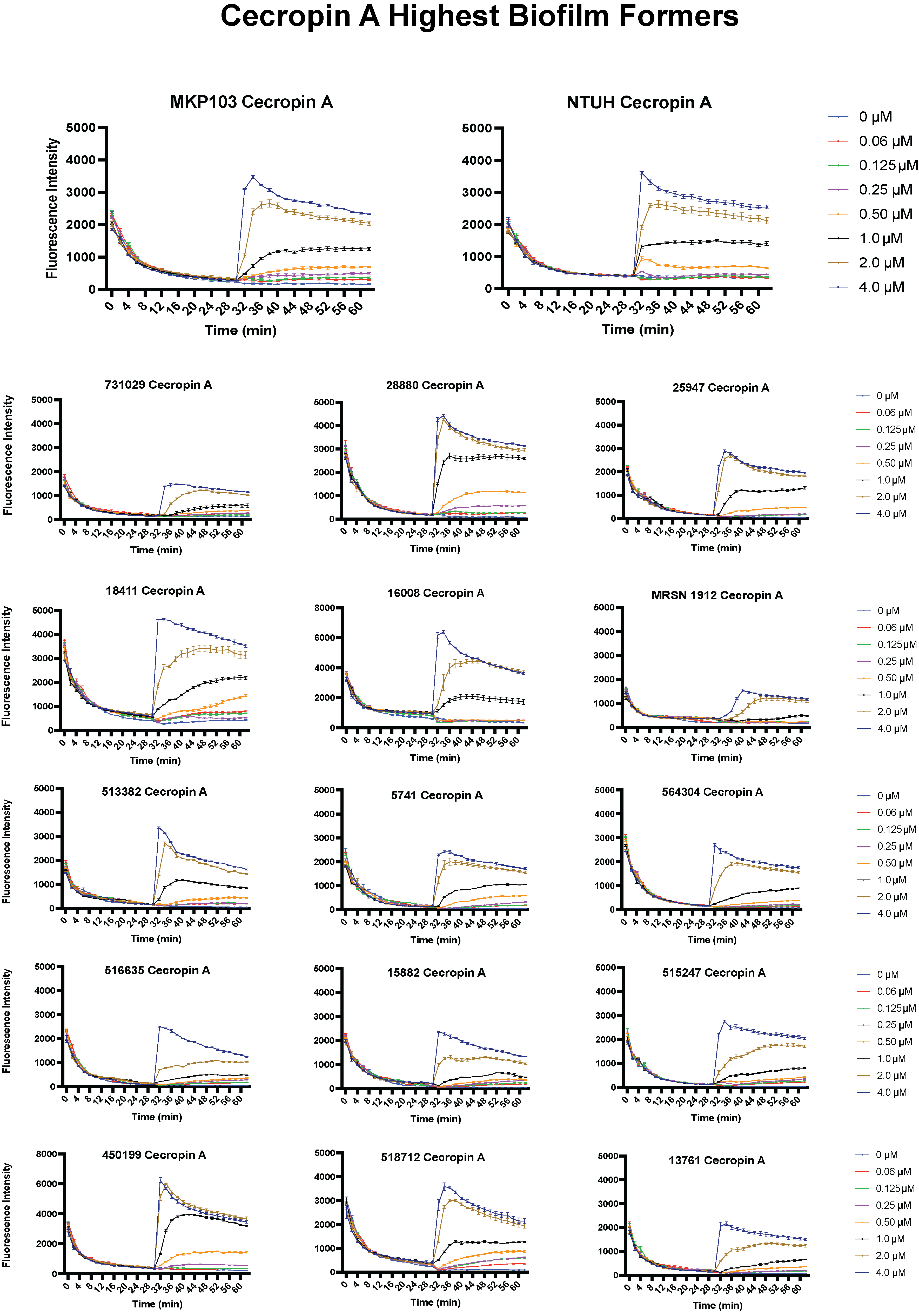

Supplement: S9 Fig — Kinetic depolarization of hvKp (NTUH K2044) and cKp (MKP103) control strains as well as the highest biofilm-forming isolates in the MRSN diversity panel are shown after treatment with cecropin A for 30 minutes. DiSC3(5) (3,3’-Dipropylthiadicarbocyanine Iodide) cationic hydrophobic dye was used. Readouts were measured in fluorescence intensity and normalized fluorescence intensity values were achieved by subtracting blank wells in 96-well plate from sample wells. Errors were reported as ±SEM. (TIF) [file ppat.1013437.s009.tif]

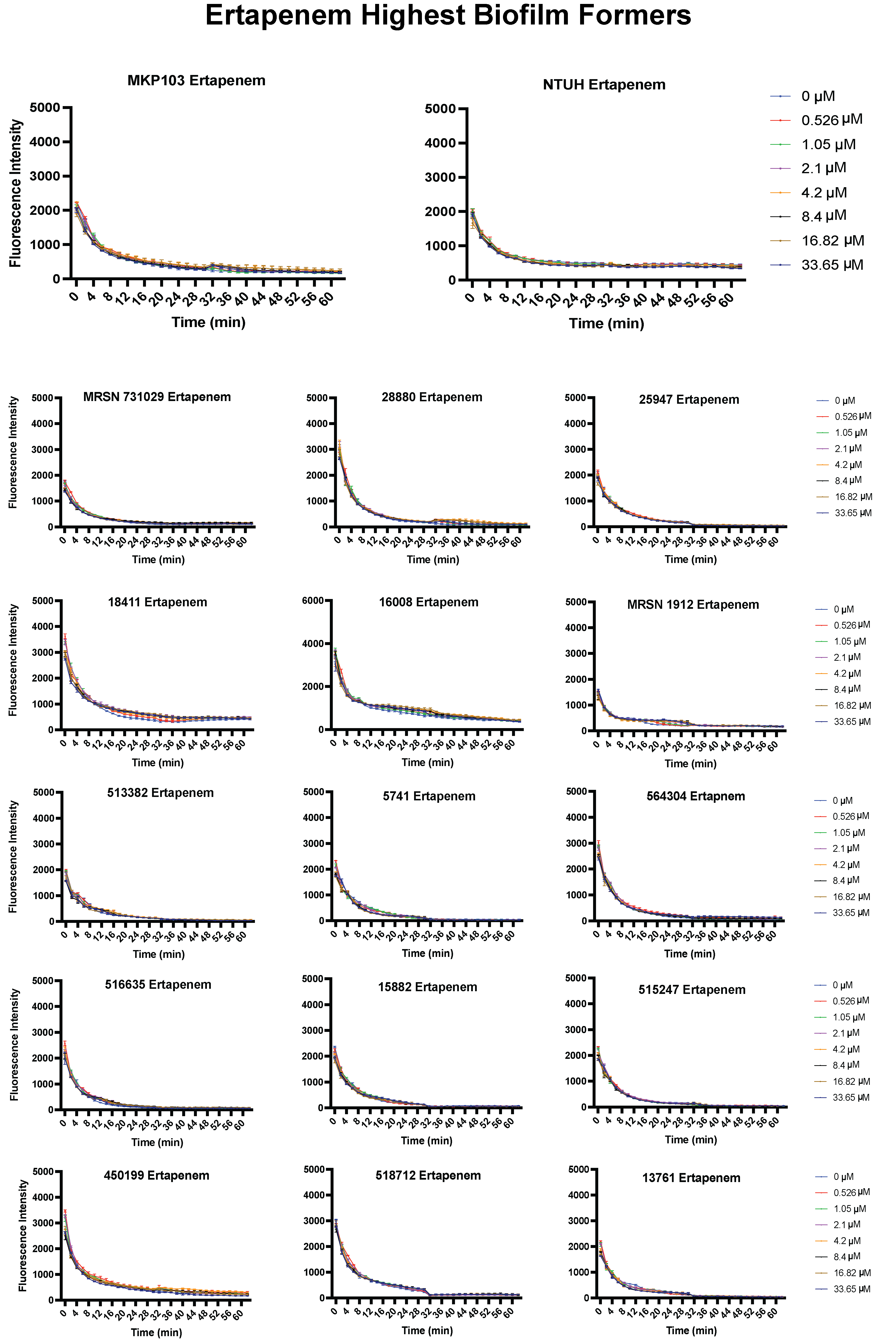

Supplement: S10 Fig — This Fig shows the depolarization of both cKp (MKP103) and hvKp (NTUH K2044) control strains and MRSN isolates with the highest biofilm formation abilities after 30-minute treatment with ertapenem. The cationic hydrophobic DiSC3 (3,3’-dipropylthiadicarbocyanine Iodide) dye was utilized. Normalized fluorescence intensity values were achieved by subtracting blank wells of 96-well plate with sample wells. Errors were reported as ±SEM. (TIF) [file ppat.1013437.s010.tif]

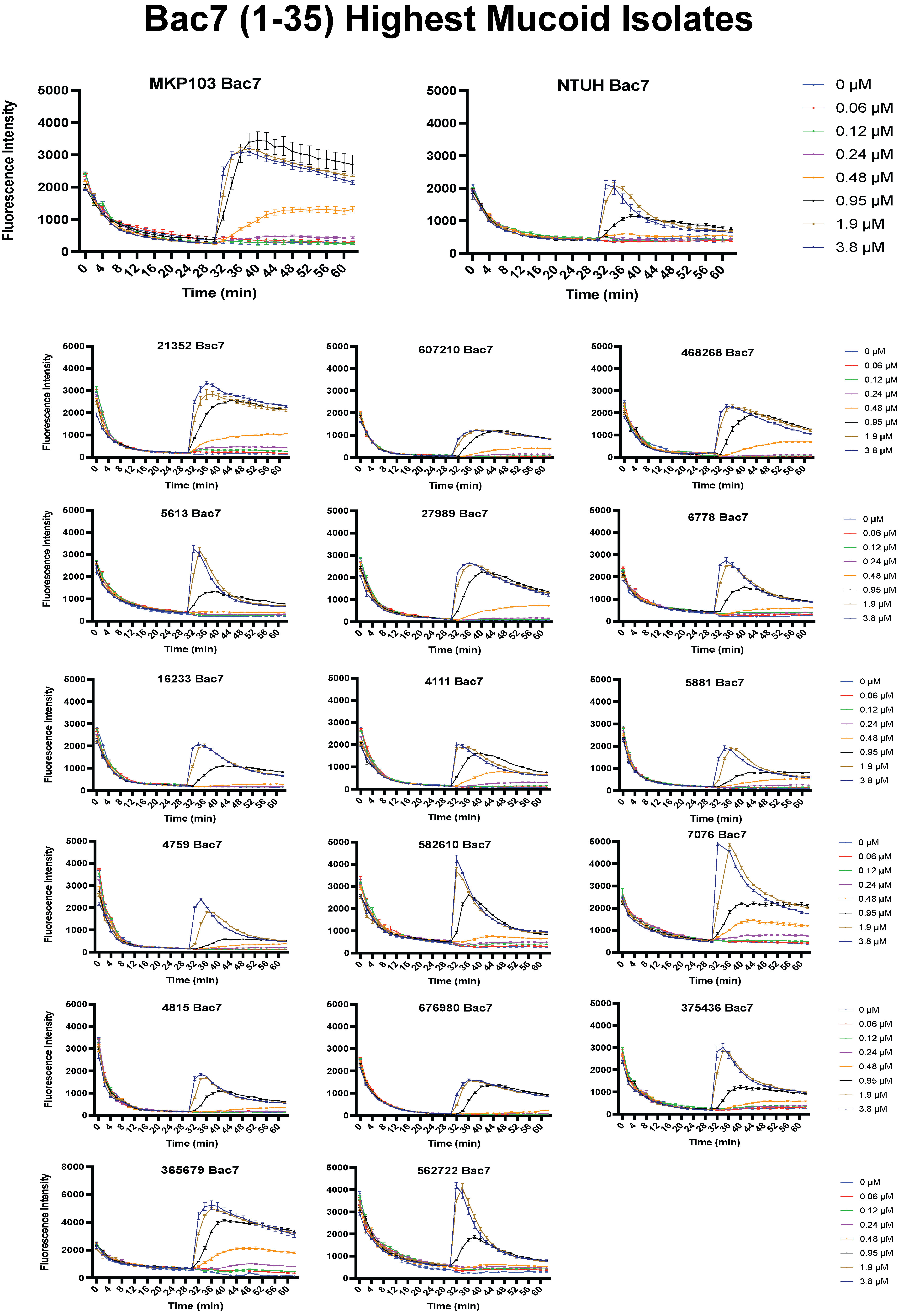

Supplement: S11 Fig — Figure shows membrane depolarization intensities after treatment with bac7 (1–35) of cKp control MKP103 and hvKp control NTUH K2044 as well as clinical isolates from the MRSN diversity panel that displayed increased %mucoviscosity. DiSC3 cationic dye was used. Readouts were measured with fluorescence intensity and were normalized by subtracting fluorescence intensity values of blank wells in 96-well plate with sample wells. Errors were reported as ±SEM. (TIF) [file ppat.1013437.s011.tif]

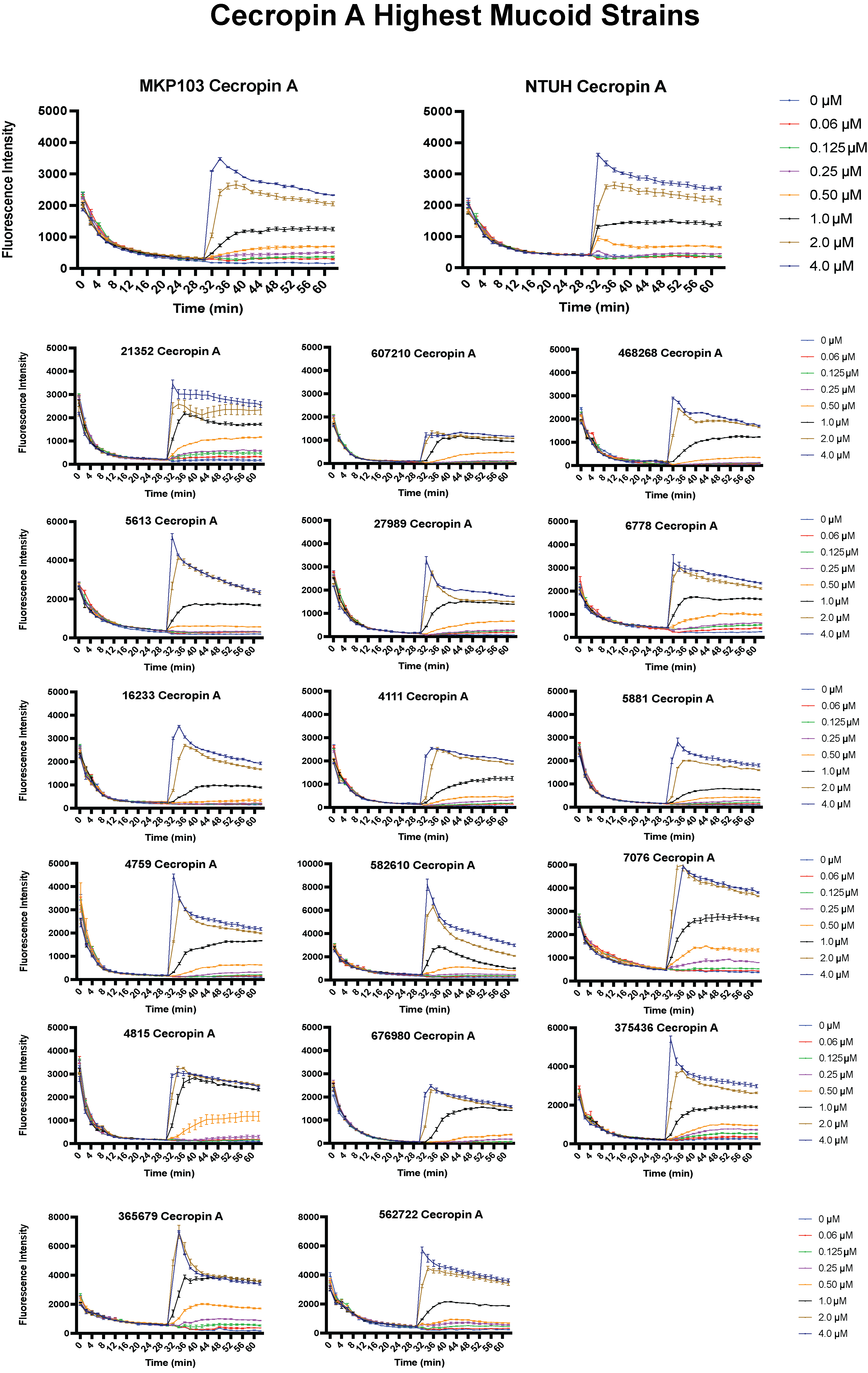

Supplement: S12 Fig — The Figure shows membrane depolarization kinetic graphs with MRSN clinical isolates treated with cecropin A that displayed the highest %mucoviscosity values in the diversity panel. cKp (MKP103) and hvKp (NTUH K2044) control strains are shown for reference. DiSC3 dye was utilized. Normalized fluorescence intensity values were achieved by subtracting blank wells in 96-well plate from sample wells. Errors were reported as ±SEM. (TIF) [file ppat.1013437.s012.tif]

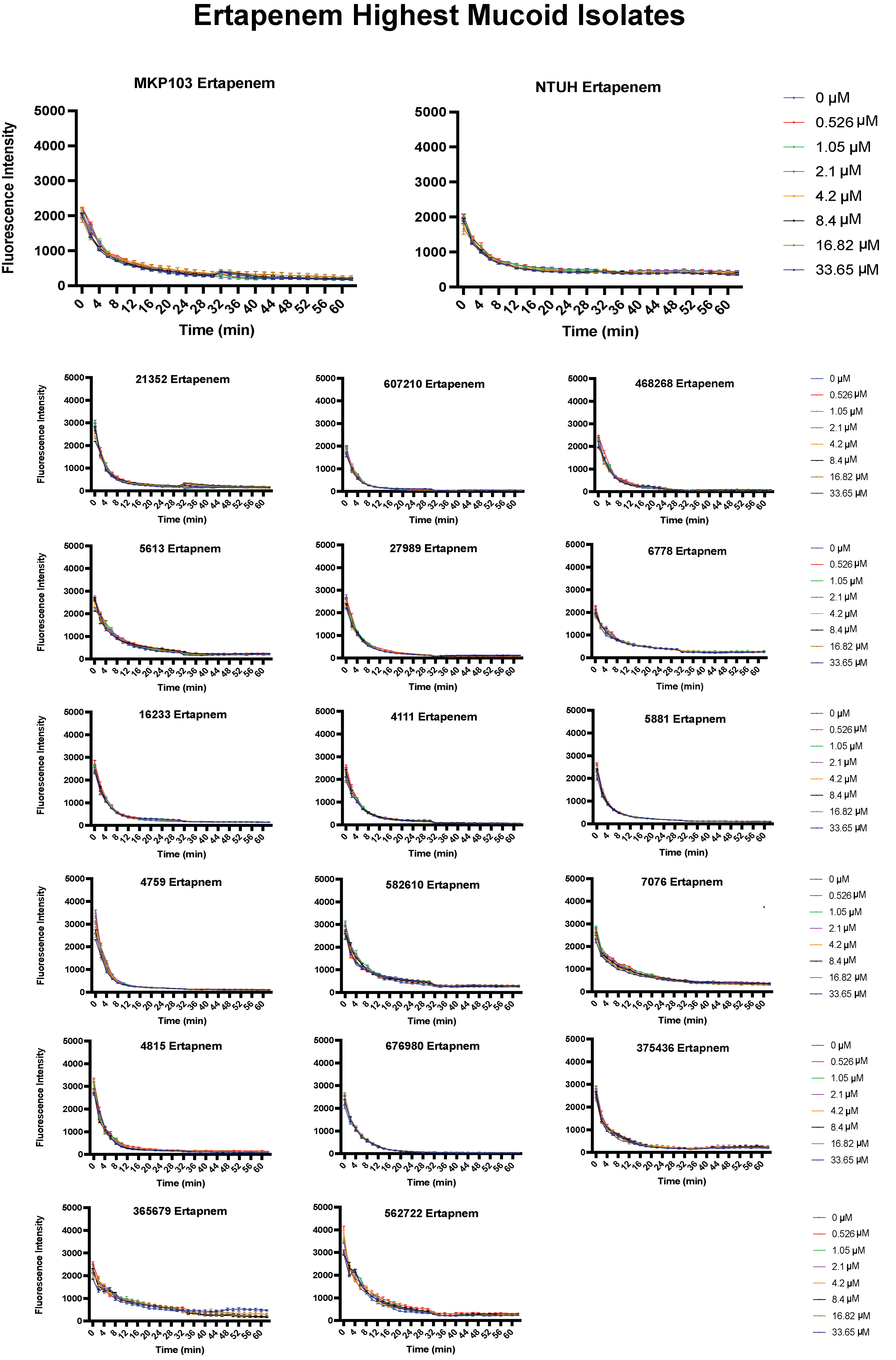

Supplement: S13 Fig — The Fig shows membrane depolarization after treatment with ertapenem in both hvKp (NTUH K2044) and cKp (MKP103) control strains as well as MRSN clinical isolates that display the highest mucoviscosity values in the MRSN diversity panel. Assays were performed with DiSC3 cationic dye. Normalized fluorescence intensity values were achieved by subtracting blank wells in 96-well plate from sample wells. Errors were reported as ±SEM. (TIF) [file ppat.1013437.s013.tif]

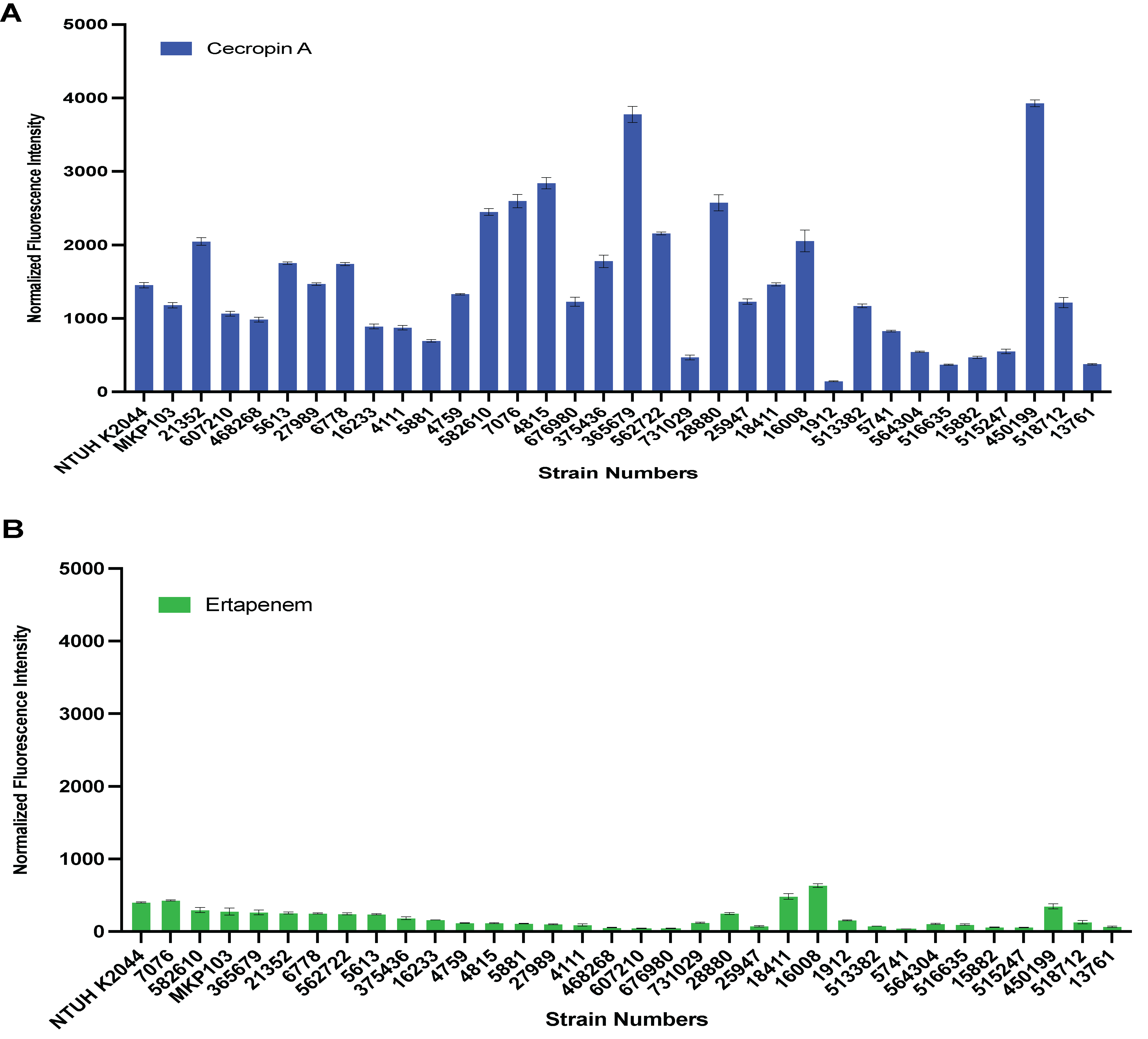

Supplement: S14 Fig — Membrane depolarization of MRSN isolates after 10 minutes treatment with 0.25 µmol L-1 cecropin A (A) or 2.1 µmol L-1 ertapenem (B). Normalized fluorescence intensity values were obtained by subtracting fluorescence intensities from the blank wells in 96-well plate from sample wells. Errors were reported as ±SEM. (TIF) [file ppat.1013437.s014.tif]

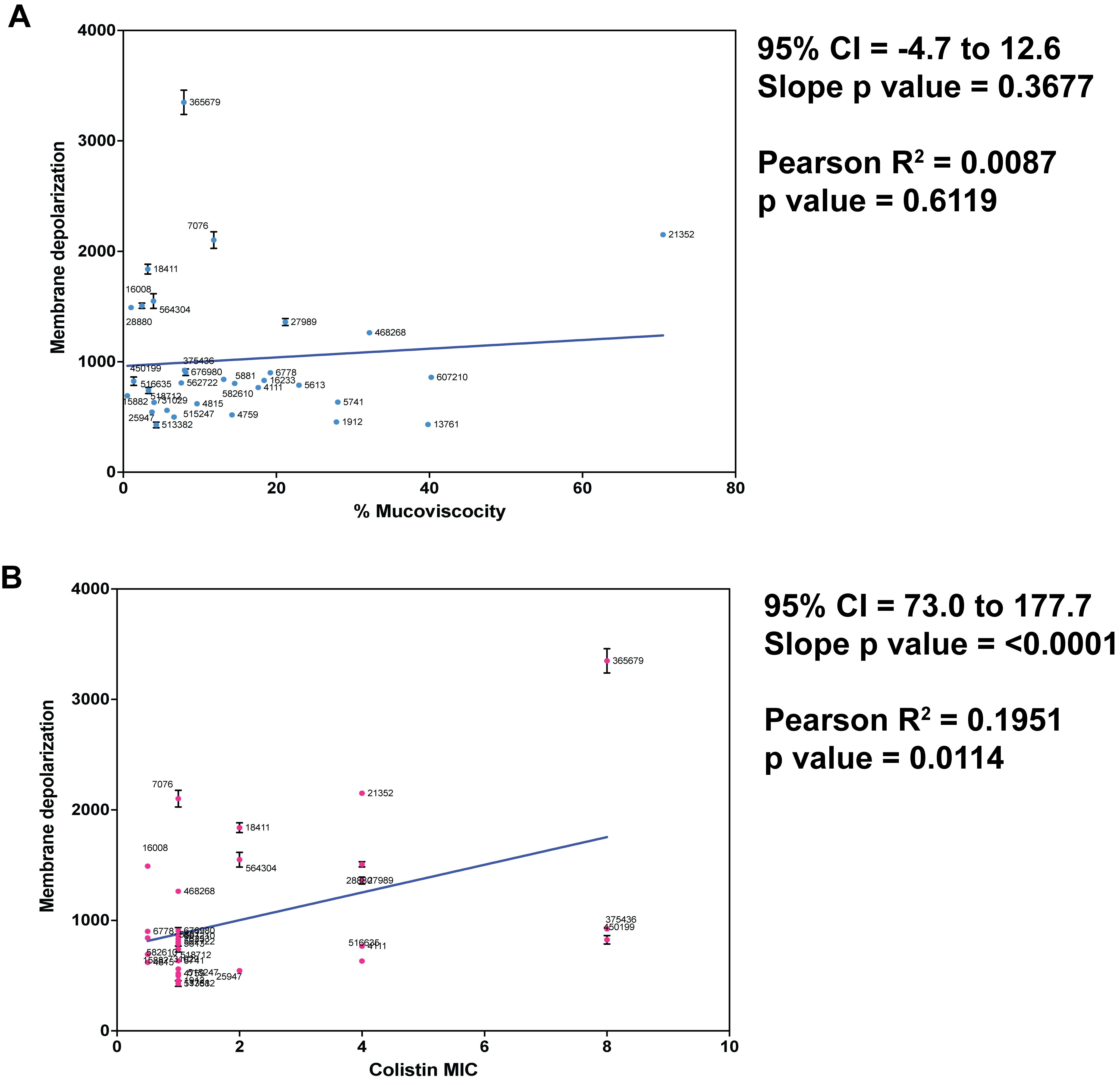

Supplement: S15 Fig — The figures show membrane depolarization of the MRSN isolates on the Y-axis and percent mucoviscosity or colistin resistance on the X-axis obtained from these isolates in our previous studies [15,66]. Simple linear regression was used to determine the slope with the 95% confidence interval and p-value of the slope shown. Correlation between the variables was determined using Pearson correlation analysis with R-squared and p-values shown. Errors for membrane depolarization were reported as ±SEM. (TIF) [file ppat.1013437.s015.tif]

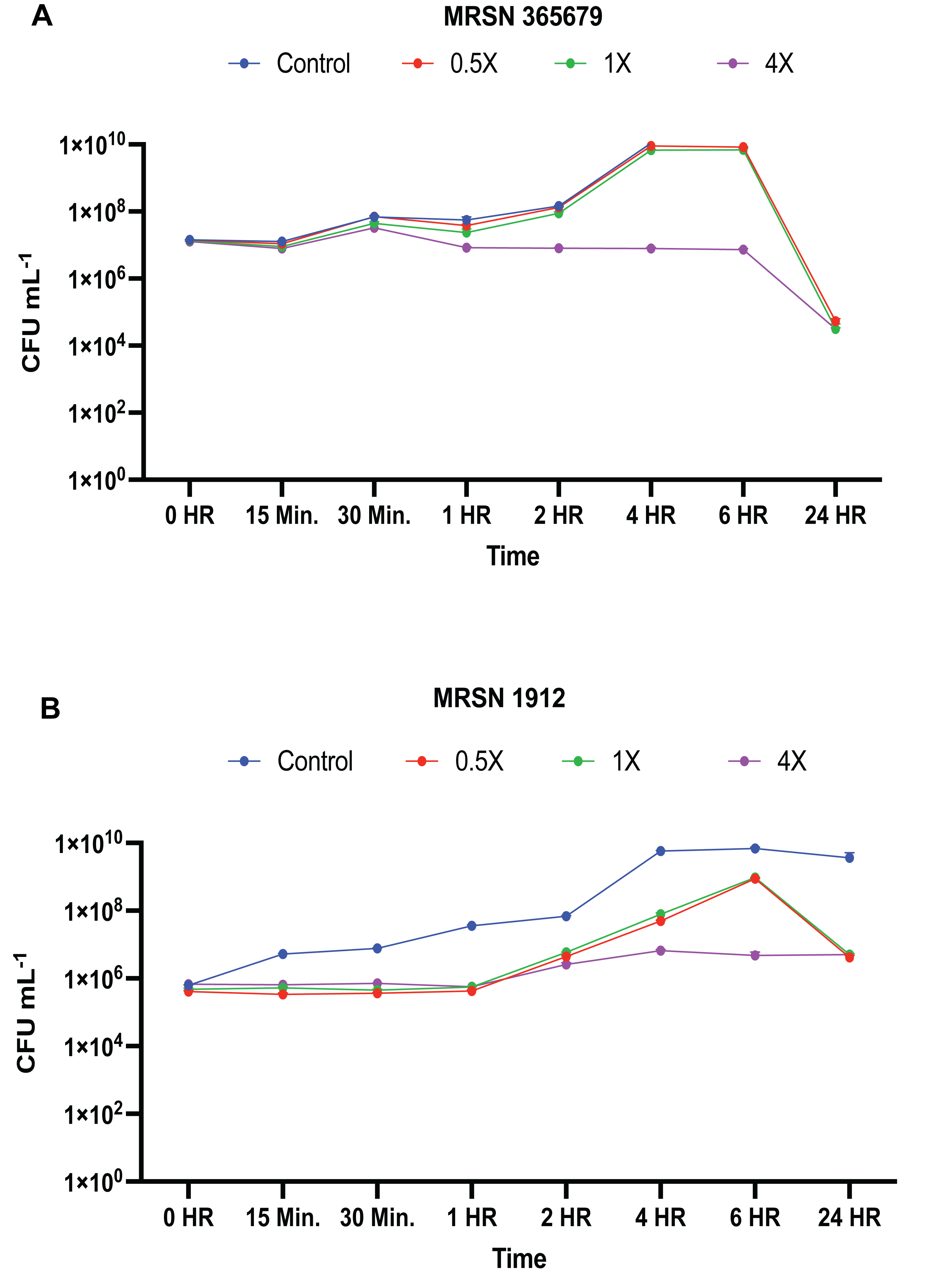

Supplement: S16 Fig — Time kill assay showing after treatment of clinical isolates MRSN 365679 (A) and 1912 (B) with bac7 (1–35) up to 24 hours. Samples were treated with 0, 0.5, 1 and 4X the minimum inhibitory concentrations (MICs), respectively with results measured in CFU mL-1. Errors were reported as ±SEM. (TIF) [file ppat.1013437.s016.tif]

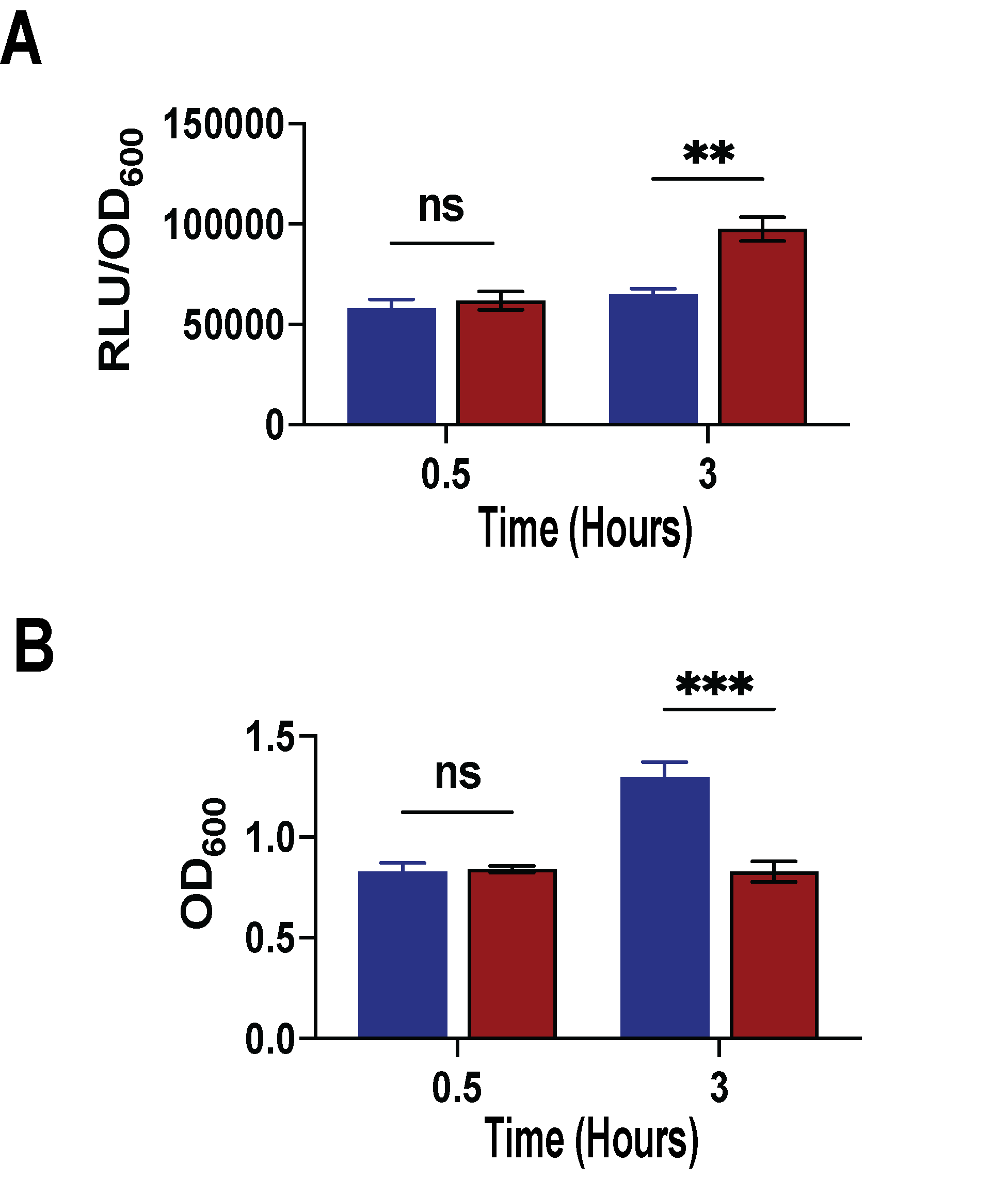

Supplement: S18 Fig — ATP quantification of MRSN 564304 biofilm embedded cells using relative luminescence units (RLU) normalized to the respective OD600 (RLU/OD600) using a CellTiter-Glo kit with no treatment and 15 µmol L-1 bac7 for 0.5 and 3 hours (A). Biofilm OD600 MRSN 564304 for each timepoint used to normalize ATP RLU (B). One-way ANOVA was used to determine significance compared to the no treatment groups for A and B with Dunnett’s correction for multiple comparisons and adjusted p-values shown (asterisks indicate p-values **** < 0.0001, ** < 0.01, and ns > 0.1) and error shown reported as ±SEM. (TIF) [file ppat.1013437.s018.tif]

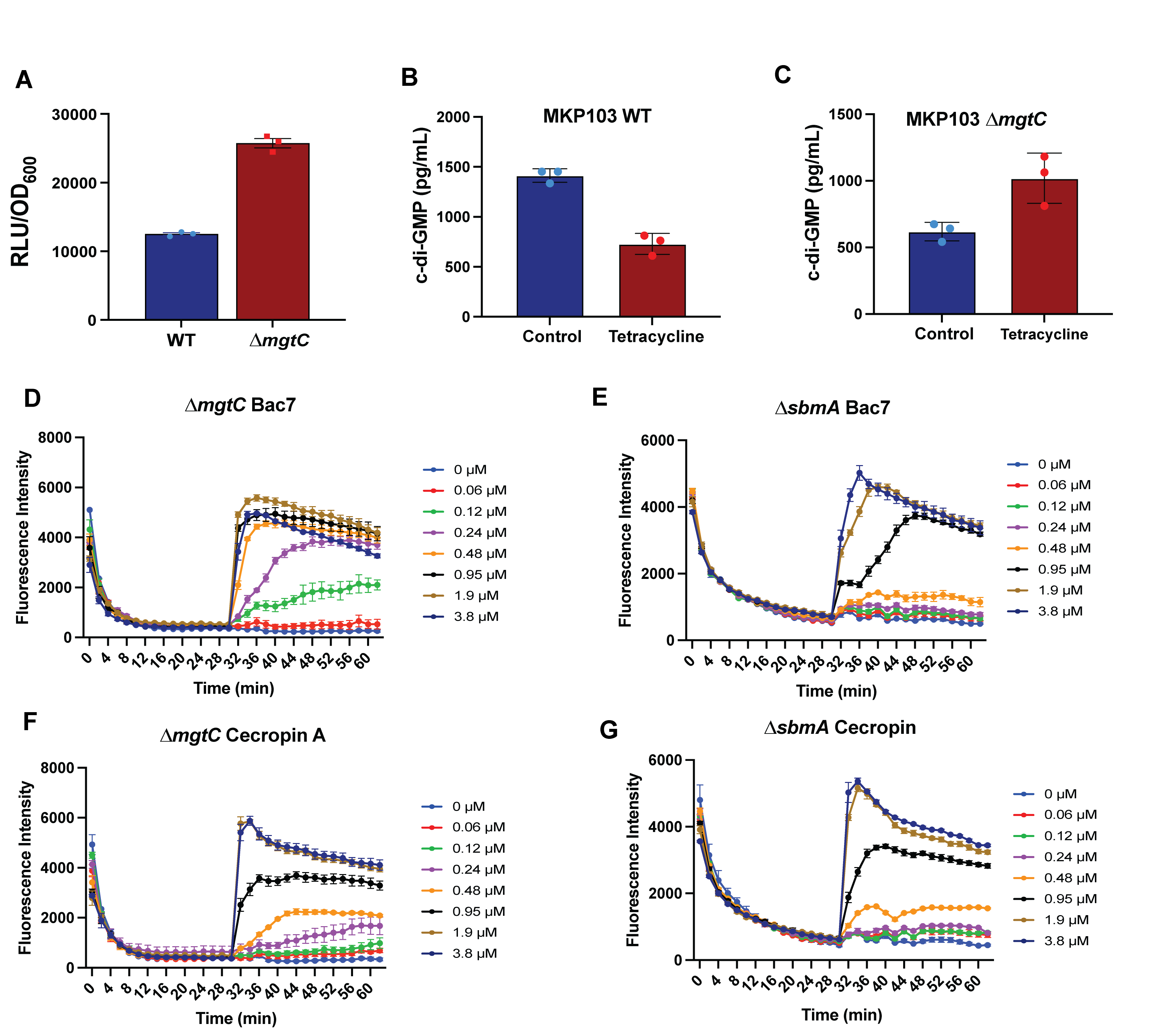

Supplement: S19 Fig — Figure shows membrane depolarization intensities after treatment with bac7 (1–35) and cecropin A positive control of MKP103 ΔmgtC and ΔsbmA transposon mutants. DiSC3(5) (3,3’-Dipropylthiadicarbocyanine Iodide) cationic dye was used. Readouts were measured with fluorescence intensity and were normalized by subtracting fluorescence intensity values of blank wells in 96-well plate with sample wells. Errors were reported as ±SEM. E and F Fig show intracellular c-di-GMP levels following treatment for 30 minutes of MKP103 WT and ΔmgtC mutant, respectively with bac7 8 µmol L-1, tetracycline and cecropin A controls using a cyclic di-GMP ELISA kit (Fig 1E). Polymyxin B resistant MKP103 and chloramphenicol transposon insert used for mutant creation lead to variable controls compared to NTUH K2044. (TIF) [file ppat.1013437.s019.tif]

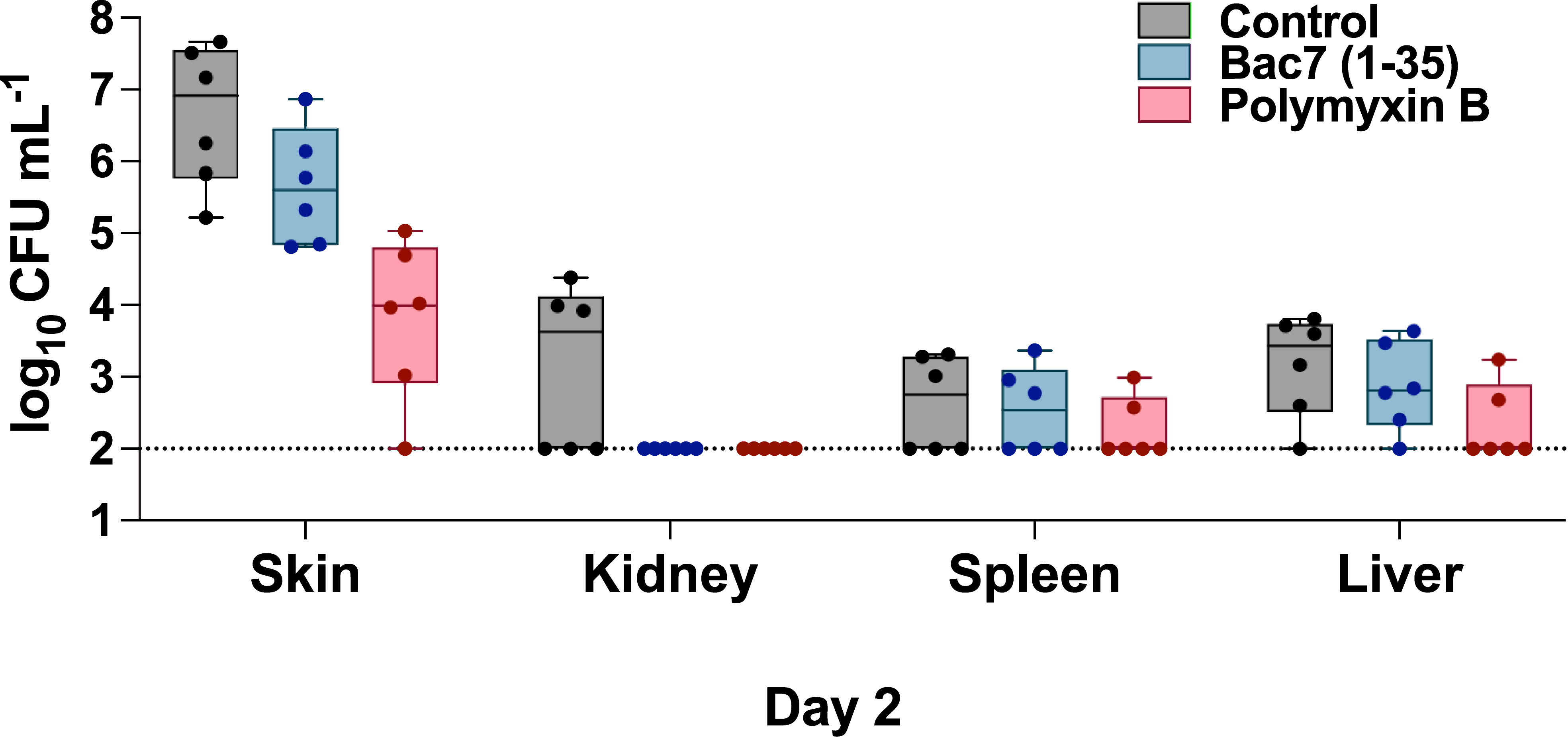

Supplement: S20 Fig — The figure shows the CFU mL-1 of the skin samples and bacteria disseminated to the kidney, spleen, and liver. Data was graphed as an interleaved scatter plot to show the mean values. Significance was determined using one-way ANOVA and p-values were obtained by comparing untreated groups to the treated groups. (TIF) [file ppat.1013437.s020.tif]
