# Supplementary material for: Molecular response to the non-lytic peptide bac7 (1–35) triggers disruption of Klebsiella pneumoniae biofilm
Source: PLoS Pathog. 2025 Dec 1;21(12):e1013437. doi: 10.1371/journal.ppat.1013437 (PMC12677791; doi:10.1371/journal.ppat.1013437)
Supplement: S1 Table — (DOCX) [file ppat.1013437.s021.docx]

**S1 Table. Kegg pathway analysis results.**

| **Pathway Identifier** | **Pathway** | **N** | **Up** | **Down** | **P.Up** | **P.Down** |
| --- | --- | --- | --- | --- | --- | --- |
| **kpu00010** | Glycolysis / Gluconeogenesis | 60 | 6 | 44 | 0.999999915 | 5.22218421079903E-08 |
| **kpu00020** | Citrate cycle (TCA cycle) | 32 | 2 | 28 | 0.999997081 | 1.57559315105521E-08 |
| **kpu00030** | Pentose phosphate pathway | 47 | 7 | 32 | 0.999923936 | 4.34339791752382E-05 |
| **kpu00040** | Pentose and glucuronate interconversions | 35 | 5 | 23 | 0.999666722 | 0.001129496 |
| **kpu00051** | Fructose and mannose metabolism | 57 | 10 | 33 | 0.999871775 | 0.002618711 |
| **kpu00052** | Galactose metabolism | 33 | 4 | 21 | 0.999861157 | 0.003332353 |
| **kpu00053** | Ascorbate and aldarate metabolism | 25 | 3 | 18 | 0.99938974 | 0.000778565 |
| **kpu00061** | Fatty acid biosynthesis | 17 | 5 | 8 | 0.853952161 | 0.322527182 |
| **kpu00071** | Fatty acid degradation | 15 | 2 | 10 | 0.993513333 | 0.027103797 |
| **kpu00130** | Ubiquinone and other terpenoid-quinone biosynthesis | 22 | 7 | 9 | 0.81481963 | 0.501133171 |
| **kpu00190** | Oxidative phosphorylation | 44 | 4 | 37 | 0.99999862 | 7.48557388172854E-10 |
| **kpu00220** | Arginine biosynthesis | 18 | 1 | 17 | 0.999860877 | 1.14075412575135E-06 |
| **kpu00230** | Purine metabolism | 85 | 23 | 45 | 0.992184475 | 0.005426345 |
| **kpu00240** | Pyrimidine metabolism | 56 | 13 | 24 | 0.995867232 | 0.312102171 |
| **kpu00250** | Alanine, aspartate and glutamate metabolism | 32 | 2 | 28 | 0.999997081 | 1.57559315105521E-08 |
| **kpu00260** | Glycine, serine and threonine metabolism | 51 | 6 | 36 | 0.999996675 | 3.91234840590598E-06 |
| **kpu00261** | Monobactam biosynthesis | 11 | 3 | 8 | 0.865703147 | 0.024111409 |
| **kpu00270** | Cysteine and methionine metabolism | 55 | 8 | 35 | 0.999982568 | 0.000159975 |
| **kpu00280** | Valine, leucine and isoleucine degradation | 16 | 1 | 9 | 0.999626079 | 0.121015219 |
| **kpu00290** | Valine, leucine and isoleucine biosynthesis | 20 | 2 | 13 | 0.999286099 | 0.015913282 |
| **kpu00300** | Lysine biosynthesis | 18 | 4 | 13 | 0.959940981 | 0.004201278 |
| **kpu00310** | Lysine degradation | 23 | 1 | 19 | 0.999988278 | 2.12922906457939E-05 |
| **kpu00330** | Arginine and proline metabolism | 33 | 5 | 23 | 0.999288773 | 0.000311049 |
| **kpu00332** | Carbapenem biosynthesis | 2 | 1 | 1 | 0.62644356 | 0.625965025 |
| **kpu00340** | Histidine metabolism | 13 | 0 | 12 | 1 | 9.64354447561342E-05 |
| **kpu00350** | Tyrosine metabolism | 26 | 3 | 21 | 0.99959899 | 1.47720946709938E-05 |
| **kpu00360** | Phenylalanine metabolism | 31 | 2 | 17 | 0.99999535 | 0.051179221 |
| **kpu00361** | Chlorocyclohexane and chlorobenzene degradation | 4 | 2 | 1 | 0.505335524 | 0.8601674 |
| **kpu00362** | Benzoate degradation | 38 | 10 | 14 | 0.963948552 | 0.658851571 |
| **kpu00364** | Fluorobenzoate degradation | 8 | 5 | 1 | 0.156590184 | 0.980485744 |
| **kpu00380** | Tryptophan metabolism | 13 | 2 | 8 | 0.984691024 | 0.082959295 |
| **kpu00400** | Phenylalanine, tyrosine and tryptophan biosynthesis | 25 | 5 | 16 | 0.987509707 | 0.009411303 |
| **kpu00401** | Novobiocin biosynthesis | 5 | 0 | 5 | 1 | 0.008809142 |
| **kpu00410** | beta-Alanine metabolism | 13 | 0 | 6 | 1 | 0.391410158 |
| **kpu00430** | Taurine and hypotaurine metabolism | 6 | 0 | 3 | 1 | 0.431580204 |
| **kpu00440** | Phosphonate and phosphinate metabolism | 8 | 2 | 1 | 0.881575993 | 0.980485744 |
| **kpu00450** | Selenocompound metabolism | 16 | 1 | 10 | 0.999626079 | 0.047479358 |
| **kpu00460** | Cyanoamino acid metabolism | 8 | 2 | 3 | 0.881575993 | 0.660004111 |
| **kpu00470** | D-Amino acid metabolism | 16 | 2 | 10 | 0.995802443 | 0.047479358 |
| **kpu00480** | Glutathione metabolism | 27 | 6 | 19 | 0.979672341 | 0.000874229 |
| **kpu00500** | Starch and sucrose metabolism | 70 | 8 | 50 | 0.999999945 | 2.68971714249558E-08 |
| **kpu00511** | Other glycan degradation | 2 | 0 | 2 | 1 | 0.150790992 |
| **kpu00520** | Amino sugar and nucleotide sugar metabolism | 50 | 9 | 31 | 0.999614829 | 0.000730487 |
| **kpu00521** | Streptomycin biosynthesis | 9 | 3 | 4 | 0.746711173 | 0.488146262 |
| **kpu00523** | Polyketide sugar unit biosynthesis | 2 | 0 | 2 | 1 | 0.150790992 |
| **kpu00525** | Acarbose and validamycin biosynthesis | 2 | 0 | 2 | 1 | 0.150790992 |
| **kpu00540** | Lipopolysaccharide biosynthesis | 30 | 13 | 13 | 0.372161454 | 0.370473158 |
| **kpu00541** | O-Antigen nucleotide sugar biosynthesis | 18 | 1 | 14 | 0.999860877 | 0.000888981 |
| **kpu00542** | O-Antigen repeat unit biosynthesis | 1 | 1 | 0 | 0.388769321 | 1 |
| **kpu00543** | Exopolysaccharide biosynthesis | 8 | 2 | 3 | 0.881575993 | 0.660004111 |
| **kpu00550** | Peptidoglycan biosynthesis | 25 | 9 | 11 | 0.687380928 | 0.367695253 |
| **kpu00552** | Teichoic acid biosynthesis | 3 | 3 | 0 | 0.058704979 | 1 |
| **kpu00561** | Glycerolipid metabolism | 25 | 3 | 16 | 0.99938974 | 0.009411303 |
| **kpu00562** | Inositol phosphate metabolism | 15 | 3 | 7 | 0.967265459 | 0.354398198 |
| **kpu00564** | Glycerophospholipid metabolism | 30 | 12 | 15 | 0.518800558 | 0.142542731 |
| **kpu00565** | Ether lipid metabolism | 1 | 0 | 0 | 1 | 1 |
| **kpu00592** | alpha-Linolenic acid metabolism | 3 | 0 | 2 | 1 | 0.335317373 |
| **kpu00600** | Sphingolipid metabolism | 3 | 0 | 3 | 1 | 0.058527801 |
| **kpu00620** | Pyruvate metabolism | 72 | 10 | 55 | 0.999999358 | 7.44370319087786E-11 |
| **kpu00621** | Dioxin degradation | 4 | 2 | 0 | 0.505335524 | 1 |
| **kpu00622** | Xylene degradation | 10 | 5 | 2 | 0.338891917 | 0.946325705 |
| **kpu00623** | Toluene degradation | 6 | 2 | 3 | 0.748995943 | 0.431580204 |
| **kpu00625** | Chloroalkane and chloroalkene degradation | 4 | 1 | 3 | 0.860525085 | 0.165981545 |
| **kpu00626** | Naphthalene degradation | 4 | 1 | 3 | 0.860525085 | 0.165981545 |
| **kpu00627** | Aminobenzoate degradation | 9 | 4 | 1 | 0.489135802 | 0.988076534 |
| **kpu00630** | Glyoxylate and dicarboxylate metabolism | 44 | 4 | 33 | 0.99999862 | 1.08045784015332E-06 |
| **kpu00633** | Nitrotoluene degradation | 3 | 0 | 3 | 1 | 0.058527801 |
| **kpu00640** | Propanoate metabolism | 38 | 1 | 26 | 0.999999993 | 0.000202288 |
| **kpu00643** | Styrene degradation | 5 | 2 | 2 | 0.643460934 | 0.642765629 |
| **kpu00650** | Butanoate metabolism | 44 | 3 | 30 | 0.999999853 | 7.2443054290227E-05 |
| **kpu00660** | C5-Branched dibasic acid metabolism | 15 | 0 | 10 | 1 | 0.027103797 |
| **kpu00670** | One carbon pool by folate | 13 | 3 | 8 | 0.932391806 | 0.082959295 |
| **kpu00680** | Methane metabolism | 35 | 3 | 28 | 0.999991775 | 7.34405862552142E-07 |
| **kpu00730** | Thiamine metabolism | 17 | 9 | 7 | 0.172527988 | 0.512722246 |
| **kpu00740** | Riboflavin metabolism | 21 | 12 | 5 | 0.068925948 | 0.953963863 |
| **kpu00750** | Vitamin B6 metabolism | 10 | 3 | 7 | 0.814173793 | 0.046454294 |
| **kpu00760** | Nicotinate and nicotinamide metabolism | 25 | 4 | 15 | 0.996759434 | 0.025789984 |
| **kpu00770** | Pantothenate and CoA biosynthesis | 25 | 5 | 14 | 0.987509707 | 0.061099219 |
| **kpu00780** | Biotin metabolism | 19 | 5 | 9 | 0.916671407 | 0.294714586 |
| **kpu00785** | Lipoic acid metabolism | 13 | 1 | 10 | 0.998353953 | 0.006036615 |
| **kpu00790** | Folate biosynthesis | 26 | 16 | 8 | 0.015922673 | 0.853121035 |
| **kpu00860** | Porphyrin metabolism | 40 | 8 | 27 | 0.996934951 | 0.000216707 |
| **kpu00900** | Terpenoid backbone biosynthesis | 16 | 9 | 6 | 0.121683841 | 0.636032735 |
| **kpu00907** | Pinene, camphor and geraniol degradation | 6 | 0 | 4 | 1 | 0.163388914 |
| **kpu00910** | Nitrogen metabolism | 27 | 3 | 17 | 0.999737232 | 0.009438126 |
| **kpu00920** | Sulfur metabolism | 41 | 13 | 14 | 0.866447729 | 0.780632937 |
| **kpu00930** | Caprolactam degradation | 4 | 1 | 1 | 0.860525085 | 0.8601674 |
| **kpu00946** | Degradation of flavonoids | 6 | 0 | 2 | 1 | 0.748358342 |
| **kpu00970** | Aminoacyl-tRNA biosynthesis | 29 | 7 | 19 | 0.969554384 | 0.003211983 |
| **kpu00997** | Biosynthesis of various other secondary metabolites | 4 | 0 | 4 | 1 | 0.022709887 |
| **kpu00999** | Biosynthesis of various plant secondary metabolites | 4 | 0 | 3 | 1 | 0.165981545 |
| **kpu01040** | Biosynthesis of unsaturated fatty acids | 3 | 2 | 1 | 0.335875289 | 0.77128885 |
| **kpu01053** | Biosynthesis of siderophore group nonribosomal peptides | 13 | 4 | 4 | 0.810153161 | 0.809368969 |
| **kpu01100** | Metabolic pathways | 1095 | 223 | 657 | 1 | 6.6503217665079E-58 |
| **kpu01110** | Biosynthesis of secondary metabolites | 391 | 75 | 251 | 1 | 4.70302025592242E-26 |
| **kpu01120** | Microbial metabolism in diverse environments | 354 | 53 | 241 | 1 | 8.05596219031562E-31 |
| **kpu01200** | Carbon metabolism | 129 | 15 | 98 | 1 | 4.08860874485351E-18 |
| **kpu01210** | 2-Oxocarboxylic acid metabolism | 42 | 0 | 35 | 1 | 3.58733559036971E-09 |
| **kpu01212** | Fatty acid metabolism | 26 | 6 | 14 | 0.97236278 | 0.086329193 |
| **kpu01220** | Degradation of aromatic compounds | 40 | 12 | 17 | 0.908347834 | 0.372776871 |
| **kpu01230** | Biosynthesis of amino acids | 145 | 21 | 103 | 1 | 1.64028311000671E-15 |
| **kpu01232** | Nucleotide metabolism | 61 | 21 | 23 | 0.801360831 | 0.620060677 |
| **kpu01240** | Biosynthesis of cofactors | 171 | 57 | 82 | 0.945460295 | 0.008489694 |
| **kpu01250** | Biosynthesis of nucleotide sugars | 43 | 11 | 26 | 0.97727253 | 0.003214163 |
| **kpu01501** | beta-Lactam resistance | 25 | 4 | 15 | 0.996759434 | 0.025789984 |
| **kpu01502** | Vancomycin resistance | 7 | 0 | 5 | 1 | 0.085295603 |
| **kpu01503** | Cationic antimicrobial peptide (CAMP) resistance | 40 | 13 | 18 | 0.839718818 | 0.259040312 |
| **kpu02010** | ABC transporters | 271 | 65 | 136 | 0.999999966 | 6.38334232168823E-05 |
| **kpu02020** | Two-component system | 149 | 54 | 72 | 0.77405584 | 0.010579514 |
| **kpu02024** | Quorum sensing | 99 | 26 | 42 | 0.997198788 | 0.261277857 |
| **kpu02030** | Bacterial chemotaxis | 8 | 1 | 5 | 0.980585514 | 0.156019215 |
| **kpu02040** | Flagellar assembly | 3 | 0 | 1 | 1 | 0.77128885 |
| **kpu02060** | Phosphotransferase system (PTS) | 79 | 13 | 46 | 0.999996583 | 0.000341549 |
| **kpu03010** | Ribosome | 55 | 54 | 0 | 1.53395470347893E-21 | 1 |
| **kpu03018** | RNA degradation | 18 | 5 | 9 | 0.889081836 | 0.230211261 |
| **kpu03020** | RNA polymerase | 4 | 2 | 2 | 0.505335524 | 0.504653201 |
| **kpu03030** | DNA replication | 17 | 6 | 7 | 0.704443333 | 0.512722246 |
| **kpu03060** | Protein export | 19 | 11 | 7 | 0.072712259 | 0.654681568 |
| **kpu03070** | Bacterial secretion system | 46 | 15 | 13 | 0.848218499 | 0.951100196 |
| **kpu03250** | Viral life cycle - HIV-1 | 1 | 1 | 0 | 0.388769321 | 1 |
| **kpu03410** | Base excision repair | 14 | 4 | 5 | 0.857725046 | 0.690156105 |
| **kpu03420** | Nucleotide excision repair | 9 | 0 | 5 | 1 | 0.242583918 |
| **kpu03430** | Mismatch repair | 23 | 7 | 10 | 0.852853063 | 0.398388482 |
| **kpu03440** | Homologous recombination | 27 | 11 | 11 | 0.493599502 | 0.491915008 |
| **kpu04122** | Sulfur relay system | 19 | 8 | 9 | 0.471733801 | 0.294714586 |
