# Supplementary material for: Molecular response to the non-lytic peptide bac7 (1–35) triggers disruption of Klebsiella pneumoniae biofilm
Source: PLoS Pathog. 2025 Dec 1;21(12):e1013437. doi: 10.1371/journal.ppat.1013437 (PMC12677791; doi:10.1371/journal.ppat.1013437)
Supplement: S2 Table — (DOCX) [file ppat.1013437.s022.docx]

**S2 Table. Lab strains used in this study.**

| **Strain** | **Strain information** | **Reference** |
| --- | --- | --- |
|  |  |  |
| **NTUH-K2044** | K1 capsule serotype; hypervirulent pathotype | **^1^** |
| **KPPR1S** | K2 capsule serotype; hypervirulent pathotype | **^2^** |
| **KPPR1S *∆wcaJ*** | KPPR1S capsule mutant lacking WcaJ | **^2^** |
| **MKP103** | ST258; multi-drug-resistant; colistin resistant | **^3^** |
| **MKP103 *∆mgtC*** | KPNIH1_13815-409::T30 transposon mutant | **^3^** |
| **MKP103 *∆sbmA*** | KPNIH1_05310-803::T30 transposon mutant | **^3^** |

1. Wu K-M, Li L-H, Yan J-J, Tsao N, Liao T-L, Tsai H-C, Fung C-P, Chen H-J, Liu Y-M, Wang J-T, Fang C-T, Chang S-C, Shu H-Y, Liu T-T, Chen Y-T, Shiau Y-R, Lauderdale T-L, Su I-J, Kirby R, Tsai S-F. Genome Sequencing and Comparative Analysis of

Klebsiella pneumoniae

NTUH-K2044, a Strain Causing Liver Abscess and Meningitis. J Bacteriol. 2009;191(14):4492-501. doi: 10.1128/jb.00315-09.

2. Walker KA, Treat LP, Sepúlveda VE, Miller VL, Heran Darwin K. The Small Protein RmpD Drives Hypermucoviscosity in Klebsiella pneumoniae. mBio. 2020;11(5). doi: 10.1128/mBio.01750-20.

3. Ramage B, Erolin R, Held K, Gasper J, Weiss E, Brittnacher M, Gallagher L, Manoil C, Silhavy TJ. Comprehensive Arrayed Transposon Mutant Library of Klebsiella pneumoniae Outbreak Strain KPNIH1. J Bacteriol. 2017;199(20). doi: 10.1128/jb.00352-17.
