# Supplementary material for: Molecular response to the non-lytic peptide bac7 (1–35) triggers disruption of Klebsiella pneumoniae biofilm
Source: PLoS Pathog. 2025 Dec 1;21(12):e1013437. doi: 10.1371/journal.ppat.1013437 (PMC12677791; doi:10.1371/journal.ppat.1013437)
Supplement: S3 Table — (DOCX) [file ppat.1013437.s023.docx]

**S3 Table. Clinical isolates used in this study.**

| **MRSN ID** | **Strain information** | | |
| --- | --- | --- | --- |
|  | **Isolation** | **KL-type** | **MLST-type** |
| MRSN 731029 | Human urine sample | 15 | 37 |
| MRSN 28880 | Human urine sample | 27 | 36 |
| MRSN 25947 | Human urine sample | 30 | 5447 |
| MRSN 18411 | Human urine sample | 14 | 2202 |
| MRSN 16008 | Human urine sample | 63 | 111 |
| MRSN 1912 | Perianal sample | 25 | 336 |
| MRSN 513382 | Human sample (not disclosed) | 30 | 342 |
| MRSN 5741 | Human respiratory sample | 30 | 234 |
| MRSN 564304 | Human urine sample | 62 | 348 |
| MRSN 516635 | Human sample (not disclosed) | 30 | 391 |
| MRSN 15882 | Perianal sample | 113 | 1686 |
| MRSN 515247 | Human sample (not disclosed) | 1 | 23 |
| MRSN 450199 | Human urine sample | 113 | 5449 |
| MRSN 518712 | Human sample (not disclosed) | 61 | 43 |
| MRSN 13761 | Human wound sample | 3 | 394 |
| MRSN 21352 | Human urine sample | 22 | 11 |
| MRSN 607210 | Human urine sample | 51 | 231 |
| MRSN 13761 | Human wound sample | 3 | 394 |
| MRSN 468268 | Human urine sample | 58 | 5450 |
| MRSN 5741 | Human respiratory sample | 30 | 234 |
| MRSN 1912 | Perianal sample | 25 | 336 |
| MRSN 5613 | Human urine sample | 148 | 1787 |
| MRSN 27989 | Human wound sample | 3 | 2279 |
| MRSN 6778 | Human urine sample | 3 | 1842 |
| MRSN 16233 | Human urine sample | 2 | 86 |
| MRSN 4111 | Perianal sample | 31 | 4833 |
| MRSN 5881 | Human wound sample | 62 | 48 |
| MRSN 4759 | Human urine sample | 38 | 37 |
| MRSN 582610 | Human respiratory sample | 20 | 268 |
| MRSN 7076 | Human wound sample | 25 | 5445 |
