# Supplementary material for: Molecular response to the non-lytic peptide bac7 (1–35) triggers disruption of Klebsiella pneumoniae biofilm
Source: PLoS Pathog. 2025 Dec 1;21(12):e1013437. doi: 10.1371/journal.ppat.1013437 (PMC12677791; doi:10.1371/journal.ppat.1013437)
Supplement: S4 Table — (DOCX) [file ppat.1013437.s024.docx]

**S4 Table. List of primer pairs used for RT- qPCR.**

| **Gene ID** | **Primer sequence (5′-3′)** | |
| --- | --- | --- |
|  | **Forward** | **Reverse** |
| *phoP* | ATGCCGAACTGCGAGAAA | GATGACGTCCTGTGGGTATTC |
| *mgtC* | GACAACGTATGGCCGGTTTA | CGACATCGCGCTGAGAATAA |
| *bcsA* | TGGAAGGCCAGCACAATATC | GCACCTGTTCCTCGCTATTT |
| *bcsB* | GGCTCCATCGACAACTGTATTA | TCCGGCAAGGCGATAAAG |
| *fimH* | TCACCGCAGGATCGTTAATC | CACGTCGTTATTGGCGTAGA |
| *16S** | CCAGCAGCCGCGGTAAT | TTTACGCCCAGTAATTCCGATT |
| *rpoD** | GATCTGATCACCGGTTTCGT | CTTCGTCGTCATCCATCTCTTC |
| *ftsZ** | GGACGCTTATCCATGCCAATA | GGATAATGCGACCGTGGTTAT |

* Housekeeping genes used in this study
