# Supplementary material for: Molecular response to the non-lytic peptide bac7 (1–35) triggers disruption of Klebsiella pneumoniae biofilm
Source: PLoS Pathog. 2025 Dec 1;21(12):e1013437. doi: 10.1371/journal.ppat.1013437 (PMC12677791; doi:10.1371/journal.ppat.1013437)
Supplement: Attachment — Submitted filename: Bac7 Molecular Response to Reviewers.docx [file ppat.1013437.s028.docx]

**RESPONSES TO REVIEWERS’ COMMENTS:**

We greatly appreciate the positive and constructive reviewer comments and believe that incorporating these suggestions has significantly strengthened the manuscript. Please see our specific responses (*in blue italics*) to all individual comments below:

**Reviewers' Comments:**

Reviewer's Responses to Questions

**Part I – Summary**

Please use this section to discuss strengths/weaknesses of study, novelty/significance, general execution and scholarship.

Reviewer #1:

In this manuscript by Beckman and colleagues, the authors seek to define the molecular mechanisms underpinning interactions between bac7 and K. pneumoniae biofilms. Using in vitro and in vivo approaches, the authors identify two mechanisms used by bac7 to elicit stress on K. pneumoniae. The authors support these findings through transcription analysis and elegant microscopy techniques. I particularly appreciated the thorough analysis of multiple K. pneumoniae strains and leveraging an in vivo model. My major concerns are text-based. The introduction was not broad enough to introduce the rationale of many subsequent experiments, and it was not until the Discussion that many aspects of bac7, biofilms, and ATP (for example) became clear. I also had difficulties with some terminology throughout, which is detailed in the Major Concerns section below. In summary, the authors have presented a thorough manuscript that would be improved with some alterations their descriptions of the data.

*We thank reviewer #1 for their thoughtful comments and complimenting our microscopy techniques thorough analysis. We understand the concerns about the introduction and terminology. We believe our revised manuscript is improved in these areas.*

Reviewer #2:

In this manuscript (PPATHOGENS-D-25-01948), the authors present data to begin to understand how the peptide bac7 disrupts biofilms and kills K. pneumoniae. Beginning with transcriptomics to determine changes in gene expression in response to bac7, they identified several pathways impacted and explored a couple of these with follow-up assays. They demonstrate that bac7 causes membrane depolarization but not lysis, and that it becomes internalized in a set of lab strains as well as with a cohort of clinical isolates. Use of mutants led to the identification of a transporter that partly responsible for uptake of bac7, and that MgtC protects from biofilm clearance by mitigating toxic ATP-levels. They end the study by testing the ability of bac7 to alleviate infection in a mouse model, finding that it reduced bacterial burden and prevented Klebsiella-mediated weight loss. Overall this is an important study as bac7 seems to be an effective treatment for Klebsiella biofilms and infections. While I encountered some confusion, I think, for the most part, the data are convincing and the conclusions sound.

There is a ton of data in this manuscript, and the text is a bit wordy. Personally, I think some editing and streamlining will improve the readability. In some cases, extra detail made it harder to read (minor comment #1), yet in others, I felt more detail would improve clarity (e.g. major comment #1). This comes with the caveat that a few of the assays here are outside my expertise, so this could be the reason for many of my comments below. As a side note, I would like to add my appreciation for figures that were large enough to easily evaluate!

*We are grateful for Reviewer #2 detailed review and complements on our data and figures. We have attempted to streamline some of the data for this revised manuscript without losing the main concepts. We believe this has strengthened our manuscript.*

**Part II – Major Issues: Key Experiments Required for Acceptance**

Reviewer #1:

1. Throughout the manuscript, the authors use the terms “biofilm dispersal” and “dissemination”. As someone familiar with the term dissemination but not as familiar with the idea of dispersal, I found this to be confusing. It seems that biofilm dispersal would be a form of dissemination since the bacteria would be breaking free of the matrix and therefore able to spread across sites. However, I think the authors use this term to imply the biofilms are disrupted – which means they can be more easily cleared by the immune system and then not disseminate. Can the authors clarify these terms somewhere in the text? Alternatively, they could use another term such as “biofilm disruption” but the authors should decide which is more appropriate given their expertise.

*We agree that differentiation between discussion of biofilm dispersal and in vivo dissemination need increased clarification in the text so that it is not misleading. Dispersal of the biofilm is the natural last step of the biofilm life cycle. These cells break from the biofilm and carry along with them the matrix material that encases them in the biofilm. We have shown in previous publications that with bac7 treatment cells released from the biofilm do not have this associated matrix, which we hypothesize will make them more sensitive to the immune system. This may actually decrease their ability to disseminate the infection to distant vital organs. Our observations with peptide treatment are not entirely consistent with the natural process of dispersal and, therefore, we have changed the terminology of ‘dispersal’ to ‘disruption’ in the revised text.*

*The in vivo experimentation performed was to evaluate bacterial colonization of distant organs. However, as the reviewer points out, bacterial barcoding would be needed for specific measures of dissemination dynamics. We have changed the text to now reflect how the peptide prevents colonization of vital organs rather than dissemination.*

a. In a similar manner for terminology, Figure 6C shows that bac7 decreases initial site K. pneumoniae in the wound model but it does not necessarily show that bac7 decreases the process of dissemination. Dissemination is an active process in which bacteria migrate across host barriers to access secondary sites. In Figure 6C, it’s possible that bac7 decreased initial site CFU to a level in which dissemination was ineffective. When there are differences in initial site CFU, bacterial barcoding is required to understand differences in dissemination dynamics (PMID34636322). The authors should clarify they did not specifically measure dissemination in the present study, they measured colonization of primary and secondary sites.

*Please see above. In addition to changing our terminology from dissemination to colonization of vital organs, we address this distinction in the discussion including the goal to specifically measure differences in dissemination dynamics in future work. We appreciate the reviewer providing references to this barcoding dissemination dynamics and have included this reference in our manuscript.*

2. The Introduction does not provide sufficient background for someone not in the biofilm field to understand the main text panels. A lot of this information is in the Discussion, but it would be helpful if it were moved to the Introduction section.

*We have added additional information on biofilm formation to the introduction. We hope this increases the clarity of our findings shown in the manuscript.*

a. As someone familiar with K. pneumoniae pathogenesis, but not in the context of biofilms, I am not sure why ATP and cyclic-do-GMP levels are important for biofilms aside from being involved in general cell signaling.

*We have added text to the introduction to clarify the role of c-di-GMP in allosteric activation of biofilm components. In addition, we have added references that have shown the importance of c-di-GMP for biofilm formation in multiple bacterial genera.*

b. In the Introduction, bac7 is suddenly introduced in Line 87. As someone unfamiliar with this peptide, the current set up does not sufficiently describe this. Is bac7 a neutrophil derived peptide? What does it seem to be doing in other bacterial species? Why would you hypothesize the role would be the same/different in K. pneumoniae?

*We understand the reviewer’s concern of the limited background into bac7 (1-35). To address this concern, we summarize the previous studies on this host defense peptide including the information that bac7 is produced within bovine neutrophils as a pro-peptide, the diversity of bac7’s mechanism of action in other species, and how we hypothesize there may be two mechanisms of action towards K. pneumoniae.*

c. In the Introduction, the paragraph in lines 68-79 describe various aspects of biofilms but these structures can widely vary between species. Can the authors clarify in the text when these references are specifically describing K. pneumoniae biofilms or features from other species? Also, can the authors provide a few sentences in this paragraph or the next on what is known about K. pneumoniae biofilms in general (what is the state of the field for this species)?

*We have attempted to address this concern with clarifications in the introduction. Specifically, we begin by describing aspects of biofilms in general but then focus on what is known about K. pneumoniae biofilms specifically, including the dependence on fimbriae, the impediment capsule polysaccharides may have on biofilm formation, and how some heavily capsulated isolates can form robust biofilms. The added information includes references to prior work describing K. pneumoniae biofilm formation.*

Reviewer #2:

1. There were numerous situations where it was not clear to me what aspect of the data lead to the stated interpretations. Some of these assays are new to me and my lack of familiarity meant I could not quickly understand how the authors reached a conclusion. I think a smidge more detail in the text would help. Think of this as the alt text that appears with images online. One example is with Fig. 2E. I didn’t get how it was determined the peptide had moved to the cytoplasm. I had to piece together info from the legend and opening the electronic figure because the green color in the intracellular space was not evident on my printout (yes, some of us still print!). I’d like to think that if I saw the green, it would have been immediately clear to me, but not all readers will understand the effect of merged images, and others are colorblind. A simple addition of something such as “green fluorescence in the cytosolic space indicated the peptide had translocated across the membrane” would help those of us less familiar with various assays. Similarly, in Fig. 2F, I can see what are likely the blebs, but what is the evidence of the capsular changes?

*We appreciate this concern and have tried to clarify how specific aspects of the data led to specific conclusions. We hope the manuscript is now easier to digest by the general audience. We have added more detail to explain the results throughout the text with particular focus on the areas of concern brought up about Fig. 2E and 2F.* To specifically address the concerns about the capsular changes in Fig. 2F we have modified the text and added arrows to the figure to show the membrane/capsular changes. We do not believe the capsule is lost but appears distorted. Lines 400-409 now read: *“Transmission electron microscopy of hvKp NTUH-K2044 using a phosphotungstic negative stain to visualize the capsule (dark halo around cell) and membrane following treatment with 7.5 µmol L^-1^ of bac7. This analysis revealed that although the membrane was largely intact, there was membrane blebbing (black arrows) indicating membrane stress (****Figure 3F****). Furthermore, although there was not a loss of capsule we observed changes in capsule localization (red arrows) (****Figure 3F****).”*

2. The number of DEG in response to bac7 exposure seems concerningly high. This is nearly half the genome! I think it would be worth addressing this, and perhaps ensuring the Log2FC and FDR cut-offs are consistent with similar studies (presentation of the p-values as -log10FDR is new to me and I didn’t do math to see if it is consistent with other reports I’ve seen).

*We understand the concern with the high number of DEG in response to bac7 exposure. We have addressed this explicitly in the text. “Due to the concentration of peptide being well above the minimal inhibitory concentration, there were a large number of genes that displayed significant differential expression (2,511 genes -log10(FDR) > 2) following treatment with bac7 compared to the no treatment control group, with 1,202 genes upregulated (log2 Fold Change > 1) and 1,309 genes downregulated (log2 Fold Change < -1) (Supplemental data file 1).”*

*The concentration of peptide used for this analysis was well above the minimal inhibitory concentration for the peptide to mirror the concentration at which biofilms are disrupted and to ensure molar concentration matches cell concentration as the cellular density for the RNA sequencing experiment was increased compared to the MIC .*

*For the volcano plot we used -log10 FDR for our RNA sequencing as False Discovery Rate cut-off is often used instead of simple p-valuse because the multiple statistical tests applied simultaneously increasing the probability of false positives. FDR is an adjusted p-value to control of the proportions of false positives defined as significant. We feel this was particularly important for this work, where there are a large amount of changes, as discussed above. To continue to address this point and address the minor concern mentioned below, we have added both FDR and p-values to Table 1 for all genes discussed in the text for transparency of all values obtained for transcriptional changes shown in this analysis.*

3. Fig 1F-G, is it known if the RNAP alpha subunit is not feedback regulated? If rpoB and rpoC are impacted by bac7 treatment, then RNAP components may not be a suitable loading control. Minor: The legend for G could use more detail—how quantified, normalized, etc., and I suggest replacing “RNAP” in 1F with alpha subunit.

*RNAP is commonly used as a housekeeping gene, but so are rpoB and rpoC, as the reviewer points out. Therefore, there remains the possibility that RNAP is slightly different in the presence of Bac7 (despite us not being able to detect any changes by RNA-seq). However, the use of a loading control is somewhat arbitrary and redundant as equivalent amounts of protein/cells were loaded as measured by OD/titer to each well. Therefore, because the samples are already normalized by OD prior to addition to the protein gel, this loading control could be considered somewhat superfluous, but still the best option we have. We have added the additional details of western blot methods and quantification . In addition, we have changed the labeling of Figure 1F to show alpha subunit as suggested and include the link to the antibody used for this work in the methods:* [*https://www.biolegend.com/en-gb/products/purified-anti-e-coli-rna-polymerase-alpha-antibody-14680*](https://www.biolegend.com/en-gb/products/purified-anti-e-coli-rna-polymerase-alpha-antibody-14680)*.*

4. In Fig. 1H, it is really hard to see the fimbriae without zooming in on the electronic file. As this is not ideal, I suggest moving Fig. S4D to Fig. 1. This partly stems from my current question about the quantification of the westerns. The TEM are much more convincing of the decrease, but too hard to see in this main figure (even with the hi-res version).

*We appreciate the concern from the reviewer on the difficulties in visualizing the fimbriae in the TEM. To address the concerns we have increased the size of 1H and moved Fig. S4D to Fig.1I.*

5. Regarding the hypothesis that type 1 fimbriae are important for biofilms, is there evidence for this in Kleb? From what I recall, it has only been experimentally demonstrated for the type 3 fimbriae. If there are references supporting this, they should be included.

I found it curious the authors chose to leave this as a hypothesis, rather than including assays with a fim mutant to test this. I know these mutants exist in KPPR1S and may be available in the MKP103 library. Inclusion of these mutants could bring the story to full circle with the mgtC element. Alternatively, a plasmid overexpressing a diguanylate cyclase that leads to increased Fim production might similarly work.

*We appreciate the reviewer question about the role of type 3 fimbriae vs type 1 fimbriae in K. pnuemoniae biofilms. Under different conditions, various Kleb strains have been shown to utilize type 1, type 3, or even capsule in biofilms. An example of a type 1-dependent biofilm is now referenced (*[*https://pubmed.ncbi.nlm.nih.gov/18411285/*](https://pubmed.ncbi.nlm.nih.gov/18411285/)*, see Fig. 6). The components important for biofilm are complex as some conditions/strains have been shown to be type 3 dependent or capsule dependent, but at least some are type 1 dependent. Considering this variability, KPPR1S and MKP103 may not be best to follow up on the expression changes we observed in NTUH-K2044.*

*In addition to this, we hypothesize that our biofilm disruption phenotype is a combination of loss of cellulose, fimbriae, and changes to polyamine production/export as these were all significantly decreased in expression following bac7 treatment. Previous studies have independently demonstrated the importance of type 1 fimbriae, c-di-GMP, and cellulose for biofilm formation and therefore did not feel specific deletions of these factors was necessary or within the scope of this work.*

6. Regarding the enormous amount of data with the MRSN collection, I have to confess I was left unclear as to what ultimately was learned from this data, and this is in part due to my unfamiliarity with the assays. I think some streamlining of the text might help make it clearer to less informed audiences.

*We have reworked the main figures and increased the clarity of the text to describe the assays and the interpretation of the results. We bring into context our hypothesis that the membranes of these isolates with variable colistin resistance may be decreasing the rate of peptide translocation into the cell. We hope the revised MRSN data is easier to interpret with our modified figures and text.*

7. The section that begins on line 373, I was confused about the choice to use the sbmA mutant. There are a million transporters, so I was left wondering why this one? There is a reference mentioned in the discussion that this protein had been shown to transport bac7. This rationale should be included here in the Results.

*Previous research with bac7 (1-35) and other polyproline peptides has shown SbmA is the major transporter used by these peptides. We have included more detailed information about this transporter’s importance in the introduction and added text in the results. We also discuss the SbmA operon further in the discussion.*

8. Given that colistin resistance can arise from mutations within the PhoPQ system, I’m a bit concerned about the use of MKP103 in Figure 5, especially since this strain did not display the expected increase in ATP in response to bac7. I cannot remember what the mechanism of colistin resistance is in this particular strain, but whether it is dependent or independent of the PhoPQ system, this should probably be addressed.

*We agree with the reviewer that colistin resistance could play a role in the activation of mgtC considering it is in the PhoP regulon. This isolate constitutively expresses phoP and therefore mgtC is already expressed before bac7 treatment is applied. We see increased membrane depolarization of MKP103 membrane following bac7 treatment. We hypothesize this is due to the peptide penetrating more slowly into the cytosol of this colistin resistant isolate. This is why there is not the same increase in ATP following bac7 treatment with this isolate. Following other comments by the reviewer we added more clarification of our hypothesis of colistin resistance and reasoning behind the results presented in the colistin resistant isolate compared to the colistin sensitive NTUH-K2044 in this section and previous sections.*

9. I noted the choice of a one-way ANOVA to analyze the significance of the mouse data. I’m far from a stats expert, but as I understand, this is a parametric test, and mouse data is usually considered non-parametric. In addition, it is more common to report median values rather than means. I comment on this just to ask that the authors ensure an appropriate test was chosen.

*We thank the reviewer for this valuable comment. Because CFU data are typically log-normally distributed, we reanalyzed the mouse infection results after applying a log₁₀ transformation to stabilize variance and approximate normality. To rigorously assess distributional assumptions, we performed both the Shapiro–Wilk and Kolmogorov–Smirnov tests. In most groups, data were consistent with normality following transformation, while some remained non-normal, particularly at the detection limit.*

*To ensure robustness, we analyzed the datasets using both parametric (one-way ANOVA with Tukey’s post hoc test) and non-parametric (Kruskal–Wallis with Dunn’s test) approaches. Bac7 (1-35) was not found to have a significant reduction with the new statistical analyses, therefore all discussion in the text reflects this and the p-values have been changed to reflect the non-parametric approach.*

*We note that in disseminated organs, the apparent lack of significance is influenced by the detection threshold of our assay (100 CFU/mL). Several animals in the Bac7(1–35) group reached this limit, and their true bacterial loads may have been even lower. Because values below the detection limit are censored to the same baseline, this reduces apparent variance and statistical power. We have now clarified this in the Methods and figure legends. We have also updated the figures and text to report both mean ± SEM and medians, in line with common practice in vivo. Together, these revisions improve statistical rigor and transparency.*

**Part III – Minor Issues: Editorial and Data Presentation Modifications**
Please use this section for editorial suggestions as well as relatively minor modifications of existing data that would enhance clarity.

Reviewer #1:

1. In Figure 1, can the authors provide context for the concentration of bac7 used in these experiments? If so many genes are differentially regulated via RNAseq in the presence of 7.5 umol bac7, should a lower concentration have been used? Potentially, this could have illuminated specific stress pathways rather than a global stress regulator. The data from Figure 2A suggest that lower concentrations could be used to elicit similar membrane stress responses. In Figure 1H, can the authors highlight where on the pictures they are observing less fimbriae? In the current images, the thickness of the cells and surrounding area looks similar.

*We understand the concerns with the number of DEG in response to bac7 exposure and have addressed this concern in a major comment mentioned above. The concentration of peptide was used to mirror the concentration at which biofilms are disrupted which is higher than the MIC. We show that at lower concentrations the peptide is more dependent on the transporter as shown in Figure 5 (now Figure 6) and only at more elevated concentrations is there membrane mediated entrance and membrane stress. We have added comments to the text when the data is displayed in Figure 6.*

*We appreciate the reviewers concern about the fimbriae visibility in figure 1H (now figure 2H). We have increased the size of these images in this figure but have also added an arrow in the untreated sample to highlight the fimbriae in this image. With no real visible fimbriae present in the treated SEM image, we just used the arrow with the untreated sample.*

2. Figure 3A-B are difficult to read as the two time points are plotted together across multiple strains. Could the authors keep the most relevant timepoint in the main text and move the other timepoints to the supplement? For Figure 3, it is difficult for a reader to keep track of the HMV state of each strain in the text and then find it in the corresponding Main/Supplement figures. Could the authors consider a panel where maybe they plot HMV levels on one axis and the DiSC3 RFU on the other axis, and then perform a correlation between the two variables? That type of panel may be more effective in getting the point across that HMV is linked to membrane depolarization.

*We appreciate the reviewers comment and have addressed the reviewers concern in several ways.*

*1) We included a correlation analysis with simple linear regression to show the slope and used Pearson correlation analysis in Supplemental Figure 15 for both sedimentation resistance (PMID: 39702763) and colistin resistance (DOI: 10.1101/2025.05.28.65659) determined in our previous studies. We do not see a correlation with mucoidy but with colistin resistance.*

*2) Only 3 isolates in the MRSN panel have the rmp locus causing HMV, indicating the other mucoid isolates resist sedimentation due an rmp idependent factor (PMID: 39702763). Therefore we avoid using the term ‘HMV’ and use ‘mucoid’. We have marked the isolates carrying the rmp locus with asterisks in Figure 3A to clearly define which isolates have this genetic element.*

*3) To improve clarity we merged figure 3A and 3B and have ordered the isolates by colistin resistance due to the new analysis we have done in the Supplemental data.*

3. In Figure 6, why did the authors use polymyxin B and not chloramphenicol? Given the results in Figure 4, it would be really striking if bac7 and chloramphenicol worked well independently but eradicated infection when used together. I do not think this experiment is necessary for publication, but would be very compelling when used in conjunction with the evidence in Figure 4.

*Polymyxin B is a commonly used control peptide for in vivo testing of peptide efficacy due to its strong efficacious activity and clinical applications. In the peptide field polymyxins set the standard of effective in vivo eradication of multi-drug resistant organisms as colistin is a last resort therapeutic for carbapenem resistant K. pneumoniae. We have added this to the text in the results section when describing our use of this control.*

*However, we appreciate the comment by the reviewer as our future studies plan to compare the efficacy of small molecule ribosomal inhibitors to our peptide ribosomal inhibitors, as well as determine if these agents have the potential to behave synergistically in vivo.*

4. In a KPPR1 bacteremia TnSeq study, SbmA was identified as a fitness factor during bloodstream infections (PMID37463183). Can the authors comment on how SbmA may be contributing to virulence?

*We are grateful for the reviewer bringing this important work to our attention as it aligns with an ongoing study in our lab. We are currently investigating spontaneous mutations in sbmA gene that arise from serial passaging in the presence of bac7. We have added text to the discussion to bring in the context of SbmA to virulence in other bacteria.*

5. Can the authors clarify the dose of NTUH used in the mouse model? The text described it as CFU/mL but does not specific the mLs used, which would indicate the total CFU administered to the mice.

*We thank the reviewer for pointing out. We have revised the sentence adding the concentration of stocks and aliquot used to infect the wounded area.*

*In the results section: “Mice with a pre-formed linear skin abrasion were infected with 1×10^4^ colony forming units (CFU) of hvKp NTUH-K2044 (20 μL of a 5×10^5^ CFU mL^-1^ solution).”*

*In the methods section: “Bacterial cultures were grown in tryptic soy broth (TSB) media until OD 0.5 was achieved. Cells were washed twice with 1× PBS and resuspended in 1× PBS to reach a final concentration of 5×10^5^ CFU mL^-1^.”*

Reviewer #2:

1. I suggest adding all genes mentioned in the results to Table 1. I found the long list of LogFC & p-values to be distracting as I read, and this isn’t essential to understanding their relevance (can at least delete the p-value). If this info is in the table, it can be removed from the text. Also, this is probably Log2FC? That ‘2’ is pretty important.

*We have added genes to Table 1 and changed the title of Table 1 to reflect the addition of these additional genes. Table 1 title is now “Expression changes with bac7 treatment” to allow for the inclusion of all expression changes beyond just fimbriae and cellulose. We have ensured that when discussion Log2FC values in the text we include ‘2’ in all instances mentioned.*

2. In the paragraph beginning on line 171, there are several places where I got confused or felt more detail was needed. This is an important section for the subsequent experiments, and I think some editing will help clarify the logic.

*We understand the reviewers concern and have added additional details in this section of the text with specifics mentioned below.*

--Line 175, the phrase “mgtC upstream hairpin loops” is a bit confusing. Do you mean hairpins within the 5’ UTR? I suggest rephrasing this to be clearer/more specific.

*We thank the reviewer for brining this confusion to our attention and have added detail to explain this more clearly. It is not in an 5’UTR but within a small protein encoded upstream of mgtC. The text to reads “Within the PhoP regulon, the protein MgtC has been shown to not only to be activated by the response regulator PhoP, but also to self-regulate through stem-loop formation in the mgtCBR leader mRNA that contains two short open reading frames, where the presence elevated ATP increases mgtC expression.”.*

--Line 178, it is not immediately clear to my why reduced ATP synthase activity results in the depletion of c-di-GMP. Is there additional detail on how/why this happens? And is it really depleted, or just reduced?

*This connection is related to the previous work in S. enterica that described the role of MgtC for intracellular survival. We have added information to the introduction to explain the connections found in the S. enterica literature and added a new Figure 1 graphical figure to display our hypotheses of the mechanism of bac7 inducing stress and how K. pneumoniae is responding to this stress.* In addition, we have adjusted the text in this section to describe c-di-GMP as reduced rather than depleted, as this is misleading as to the fact that c-di-GMP is still present but at decreased levels.

--Line 181, “Repeating the bac7 treatment used for…” This caused me to back up and re-read a couple of times. Perhaps something like “using the same conditions as for…” would help?

*We have changed the language to that suggested by the reviewer as it now reads “Using the same bac7 treatment conditions that was used for RNA sequencing…”*

3. Lines 228-229, I think this is referring to the fluorescence intensity following bac7 (or control) treatment rather than intensity “of bac7”? I initially read this as a control for background fluorescence of the compound, but then questioned what was being referred to here. Had to look at the supplemental figure to know what this meant.

*We thank the reviewer for bringing this typo to our attention. The fluorescence discussed in this sentence refers to the membrane DiSC3 dye not the peptide. The sentence now reads “The fluorescence intensity induced by bac7 was not as high or as sustained as the positive control membrane lytic peptide cecropin A although well above the negative control non-lytic small molecule ertapenem (****Supplemental Figure 6****).”*

4. Line 244, I think this should be referencing 2E rather than 2C? I noticed a few other minor typos and assume these will be caught in revision.

*We thank the reviewer for bringing these typos to our attention. We have changed 2C in this instance to 2E and have also combed through the entire manuscript to ensure we reference the correct figures/supplemental figures throughout the manuscript.*
